# Timescale Separation of Positive and Negative Signaling Creates History-Dependent Responses to IgE Receptor Stimulation

**DOI:** 10.1038/s41598-017-15568-2

**Published:** 2017-11-14

**Authors:** Brooke Harmon, Lily A. Chylek, Yanli Liu, Eshan D. Mitra, Avanika Mahajan, Edwin A. Saada, Benjamin R. Schudel, David A. Holowka, Barbara A. Baird, Bridget S. Wilson, William S. Hlavacek, Anup K. Singh

**Affiliations:** 1 Department of Systems Biology, Sandia National Laboratories, Livermore, CA USA; 2 Department of Chemistry and Chemical Biology, Cornell University, Ithaca, NY USA; 3 Department of Biotechnology and Bioengineering, Sandia National Laboratories, Livermore, CA USA; 4 Theoretical Biology and Biophysics Group, Theoretical Division, Los Alamos National Laboratory, Los Alamos, NM USA; 5 Department of Pathology, University of New Mexico School of Medicine, Albuquerque, NM USA; 6 Biological and Material Sciences, Sandia National Laboratories, Livermore, CA USA

## Abstract

The high-affinity receptor for IgE expressed on the surface of mast cells and basophils interacts with antigens, via bound IgE antibody, and triggers secretion of inflammatory mediators that contribute to allergic reactions. To understand how past inputs (memory) influence future inflammatory responses in mast cells, a microfluidic device was used to precisely control exposure of cells to alternating stimulatory and non-stimulatory inputs. We determined that the response to subsequent stimulation depends on the interval of signaling quiescence. For shorter intervals of signaling quiescence, the second response is blunted relative to the first response, whereas longer intervals of quiescence induce an enhanced second response. Through an iterative process of computational modeling and experimental tests, we found that these memory-like phenomena arise from a confluence of rapid, short-lived positive signals driven by the protein tyrosine kinase Syk; slow, long-lived negative signals driven by the lipid phosphatase Ship1; and slower degradation of Ship1 co-factors. This work advances our understanding of mast cell signaling and represents a generalizable approach for investigating the dynamics of signaling systems.

## Introduction

Central players in inflammation and allergic reactions include mast cells and basophils, which upon stimulation with a multivalent antigen, release histamine and other inflammatory mediators in a process called degranulation. Stimulation occurs when a multivalent antigen induces aggregation of the high affinity receptor for IgE, also known as FcεRI. Receptor aggregation leads to activation of several kinases, including the protein tyrosine kinase Syk, which phosphorylates an array of downstream targets to promote degranulation. Positive signals for degranulation generated by FcεRI and Syk are held in check by negative regulatory processes^1^. The dynamic interplay between positive and negative signals influences how a cell responds to inputs. A complex input waveform, such as the concentration of an antigen that varies over time, offers a means to elucidate signaling dynamics that can give rise to seemingly enigmatic phenomena, such as desensitization.

Desensitization can arise with repeated exposure to an antigen^2–7^. A mast cell that has undergone nonspecific desensitization will show attenuated responses to an antigen it has previously encountered, as well as other antigens. Mechanisms inducing nonspecific desensitization are likely to operate at the level of receptor-proximal signaling because antigen stimulation of primary human mast cells dampens responses to an unrelated antigen, without affecting secretagogues that bypass the receptor^8^. Several proteins, including the lipid phosphatases Ship1 (Inpp5d) and PTEN and the protein tyrosine phosphatase Shp1 (Ptpn6), have been implicated in negative regulation of mast cell signaling^9^, but the molecular processes governing desensitization have not been fully characterized. This is due in part to the technical challenge of exposing cells to stimuli that change over time. However, the question of how complex inputs affect cellular outputs can now be addressed with microfluidic devices.

Microfluidic technology allows for precise manipulation of fluids at timescales of seconds. This capability can be leveraged to expose single cells to complex waveform inputs, such as pulsatile, ramp, square-wave and sinusoidal stimuli. Indeed, microfluidic devices have been used to produce periodic stimuli to measure the frequency dependence of signal processing in the osmo-adaptation pathway of yeast^10^, to quantify the bandwidth of the HOG MAP pathway in yeast^11^, and to characterize responses of amoebae to pulses of chemoattractant^12^. Similarly, microfluidic devices have been used to decode, with the aid of mathematical models, how NF-$\kappa$ B activation depends on stimulus intensity and duration^13,14^.

Here, we used a microfluidic chip to characterize the frequency response of an antigen receptor signaling system that plays an important role in immunity. We find that the frequency response properties of the system allow antigen exposure (for a finite time) to transiently desensitize cells and to prime cells for a hyperactive response upon a second exposure to antigen.

## Results

### Exposing Mast Cells to Complex Waveform Inputs

**Figure 1:**
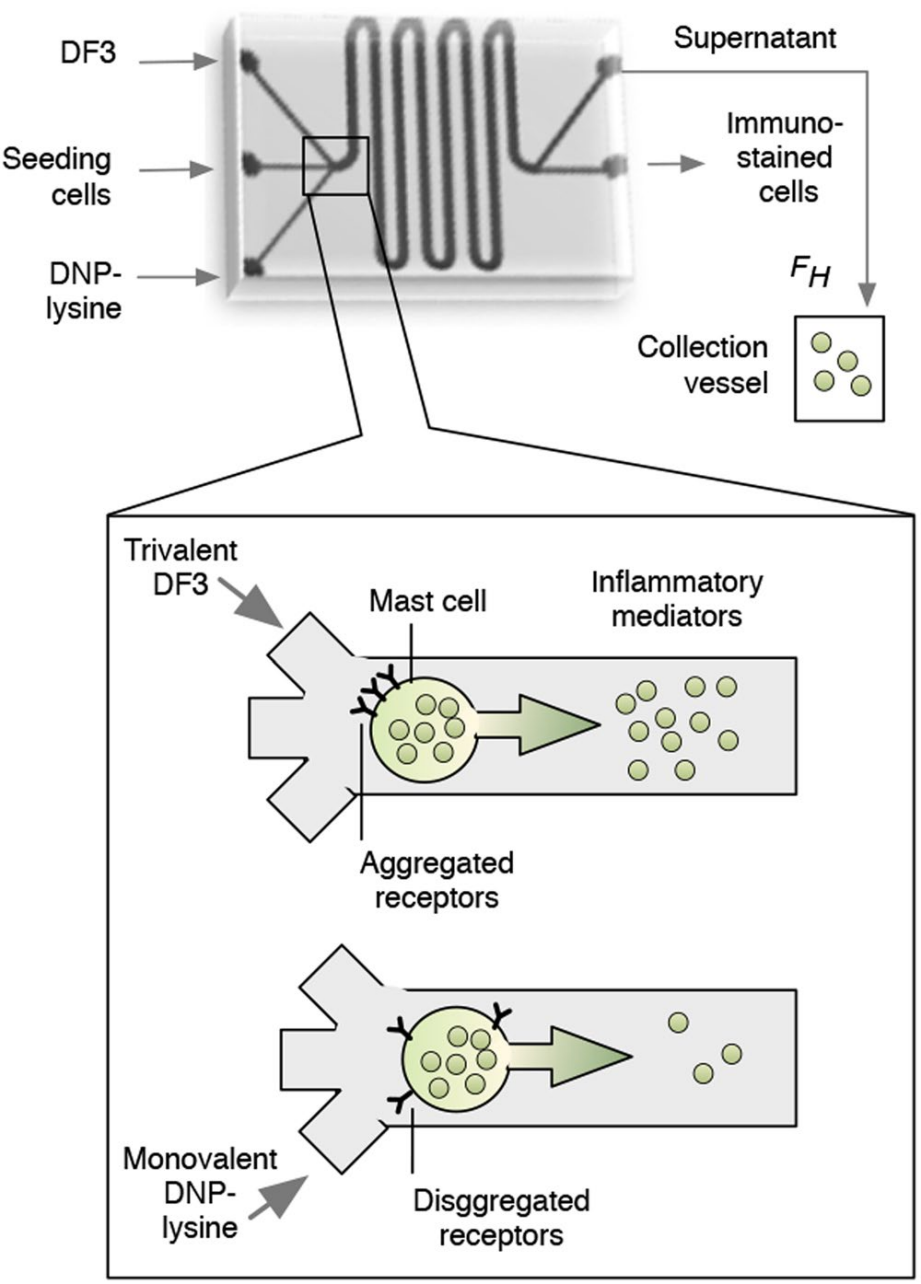
A microfluidic device for activation and deactivation of IgE receptor (FcεRI) signaling in mast cells. Top: An illustration of the microfluidic device with inlets, outlets (*F*_*H*_ is the exit flow rate), and serpentine channels. Bottom: DF3, a trivalent DNP ligand, induces aggregation of FcεRI via interaction with FcεRI-bound anti-DNP IgE, leading to release of inflammatory mediators including β-hexosaminidase. In contrast, monovalent DNP-lysine induces breakup of aggregates, thereby halting FcεRI signaling and attenuating release of inflammatory mediators.

To expose cells to alternating environments of stimulation and input quiescence, mast cells were incubated in a microfluidic device. The design of the device is illustrated in Fig. 1 (top panel). The chip has three inlets for loading cells, exchanging reagents, and buffer washing, as well as two outlets for collecting secreted material, immuno-stained cells, and waste. The channel’s serpentine design minimizes dead volume and maximizes the effective surface area for seeding of cells. The microfluidic chip is integrated with miniaturized electronic valves, optical elements, actuated pressure controllers, and data acquisition software, forming a self-contained platform that allows for precise control of the microenvironment of single cells and measurement of cellular responses to environmental perturbations. Significantly, a complete exchange of media/reagents can be accomplished in less than 20 seconds.

We used the device to expose cells to alternating flows of medium containing multivalent or monovalent ligands, which are stimulatory and non-stimulatory, respectively. A schematic of how a mast cell responds to these ligands is shown in Fig. 1 (bottom panel). As our multivalent ligand, we chose the trivalent DNP ligand, DF3, because it is symmetric, structurally well-defined, and a potent secretagogue^15^. DF3 was introduced through the microfluidic stream to crosslink DNP-specific IgE bound to cell-surface FcεRI and to initiate the intracellular signaling process. This process leads to release of inflammatory mediators within 1–2 min. A DF3 dose of 10 nM was chosen based on prior work determining that this dose is optimal for degranulation^15^. Following exposure to DF3, exposure to excess monovalent DNP-lysine causes receptor aggregates to quickly break up, resulting in reversal of intracellular signaling^16^. After ablation of receptor aggregation, DNP-lysine has no further effect because DNP-lysine does not induce FcεRI signaling (Supplementary Fig. S1). Thus, for long intervals between pulses of multivalent ligand, medium only was used after 5 min of DNP-lysine exposure, which was sufficient to disaggregate receptors and halt antigen-induced degranulation (Supplementary Fig. S2). The microfluidic device enabled us to rapidly change the environment of cells and to expose cells to abrupt pulses of stimulatory (DF3) and non-stimulatory (DNP-lysine) inputs. For example, in one protocol, we exposed cells to a 5 min pulse of DF3, followed by a 5 min pulse of DNP-lysine, and finally a second 5 min pulse of DF3. We characterized the magnitude of responses during the second DF3 pulse compared to the first. The responses that we measured were 1) cumulative amount of exocytosed β-hexosaminidase as measured by a β-hexosaminidase activity assay and 2) Syk phosphorylation at an activating site (Y346) as measured by flow cytometry^17,18^.

**Figure 2:**
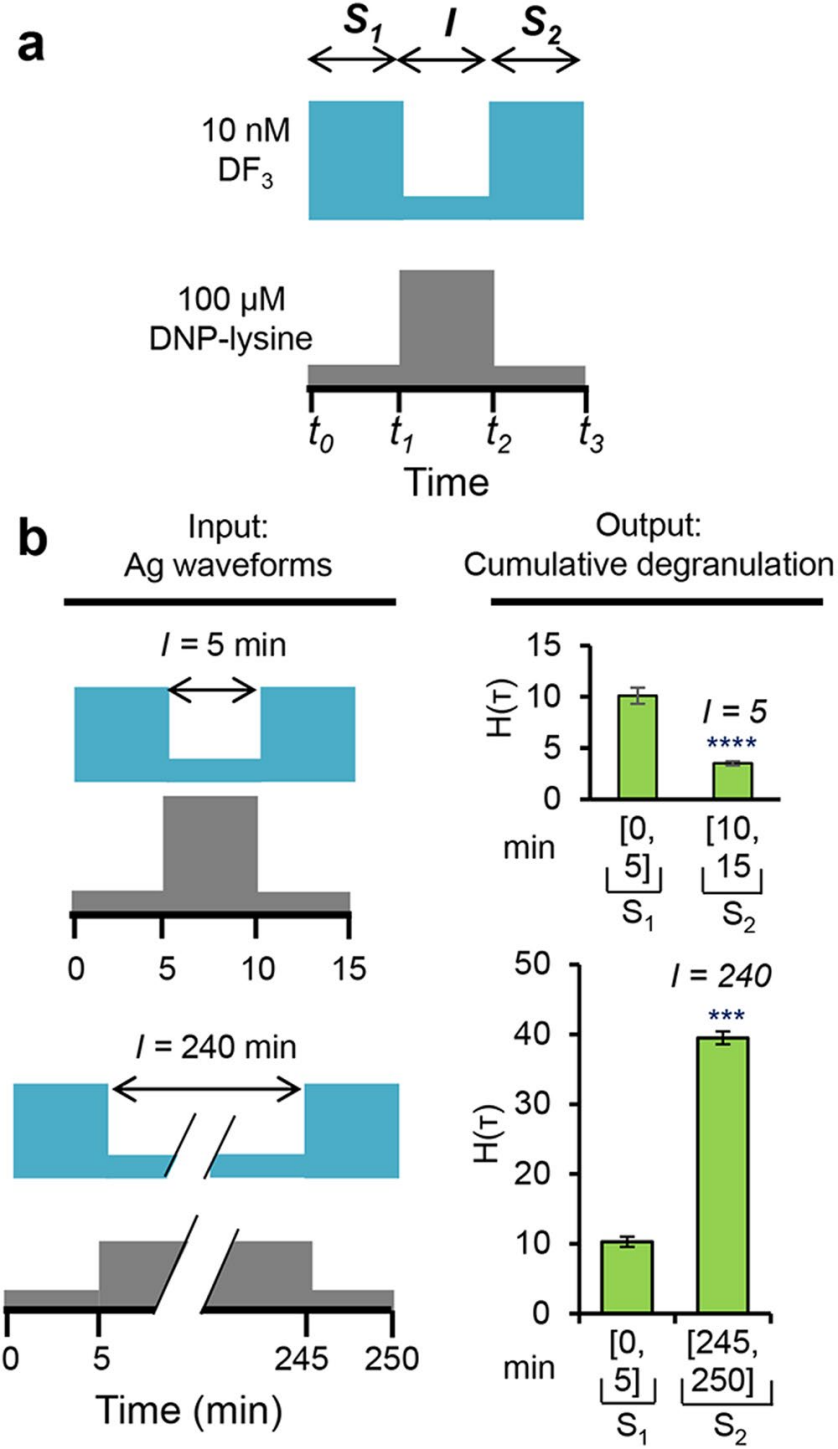
Responses to complex waveform inputs, implemented with the microfluidic device of Fig. 1. (**a**) Between times *t*_*0*_ and *t*_1_, cells were exposed to a first pulse of 10 nM DF3, designated *S*_1_. Between times *t*_*1*_ and *t*_2_, cells were exposed to 1 mM DNP-lysine. This period is designated *I*. Finally, between times *t*_2_ and *t*_3_, cells were exposed to a second pulse of 10 nM DF3, designated *S*_2_. (**b**) Responses during *S*_2_ depend on the length of *I* (compare top and bottom panels). We measured degranulation during *S*_1_ and *S*_2_, represented by *H(S*_1_) and *H*(*S*_2_). As can be seen at top right in panel b, with *I* = 5 min, *H*(*S*_2_) < *H*(*S*_1_). In contrast, as can be seen at bottom right in panel b, with *I* = 240 min, *H*(*S*_2_) > *H(S*_1_); Data are presented as mean ± S.D (n = 3), two-tailed paired *t*-test comparison of % H(_T_) values at S_2_ versus S_1_ (****P < 0.0001; ***P < 0.001; *I* = 5 min, *P* = 6.99 × 10^−5^; *I* = 240 min, *P* = 8.13 × 10^−4^).

Our nomenclature for discussing inputs and outputs is introduced in Fig. 2a. The first pulse of DF3 stimulation is designated *S*_1_. The duration of the interval of signaling quiescence (initiated by DNP-lysine exposure) is designated *I*, and the second pulse of DF3 stimulation is designated *S*_2_. The amount of degranulation/secretion occurring within a given time interval *τ*, measured as the percent of maximum degranulation, is designated *H*(*τ*). In the experiments described below, we measured *H(S*_1_), implemented a range of durations for *I*, and determined how *I* impacts *H(S*_2_).

### Mast Cells Exhibit Short- and Long-Term Memory

We initially considered an *S*_1_ duration of 5 min. Interestingly, we found that when *I* was short, e.g., 5 min, *H(S*_2_) was greatly diminished relative to *H(S*_1_) (Fig. 2b, top panel, *P* = 6.99 × 10^−5^), i.e., cells exhibited desensitization or “short-term memory.” To determine how the duration of the first pulse influences desensitization, we compared results after varying the duration of the first exposure to DF3 (30 s–5 min). Initial exposures as short as 30 s, followed by a 5 min exposure to monovalent ligand, induced desensitization to a second pulse of the trivalent ligand (Supplementary Fig. S3). This phenomenon also occurs if cells are sensitized to both dansyl (DNS) and DNP and then sequentially exposed to DNS and DNP ligands (Supplementary Fig. S4). Thus, the desensitization process found here is nonspecific and insensitive to the duration of initial antigen exposure. We also subjected cells to continuous stimulation with DF3 and found that degranulation continues to rise for as long as 10 min (Supplementary Fig. S5). Thus, desensitization does not result from depletion of the cell’s supply of β-hexosaminidase. Likewise, desensitization cannot be attributed to loss of signaling because of antigen-induced receptor internalization, which results in only a modest reduction in cell-surface receptor abundance (Supplementary Fig. S6). Instead, desensitization appears to arise from changes in the state of the FcεRI signaling network that occur upon antigen stimulation.

In contrast to the observations reported above, when *I* was long, e.g., 240 min, *H(S*_2_) was 39.5 ± 0.92%, a value much greater than that of *H(S*_1_ *)*, 10.2 ± 1.49% (Fig. 2b, bottom panel, *P* = 8.13 × 10^−4^). We term this phenomenon “long-term memory” or priming. These results show that initial desensitization is not a static condition and suggest that the signaling state within the cell changes over the course of *I* to determine the magnitude of subsequent degranulation responses. Thus, the memory phenomena we have observed are dynamical.

### Ship1 Activates Short-Term Memory and the Proteasome Activates Long-Term Memory

**Figure 3:**
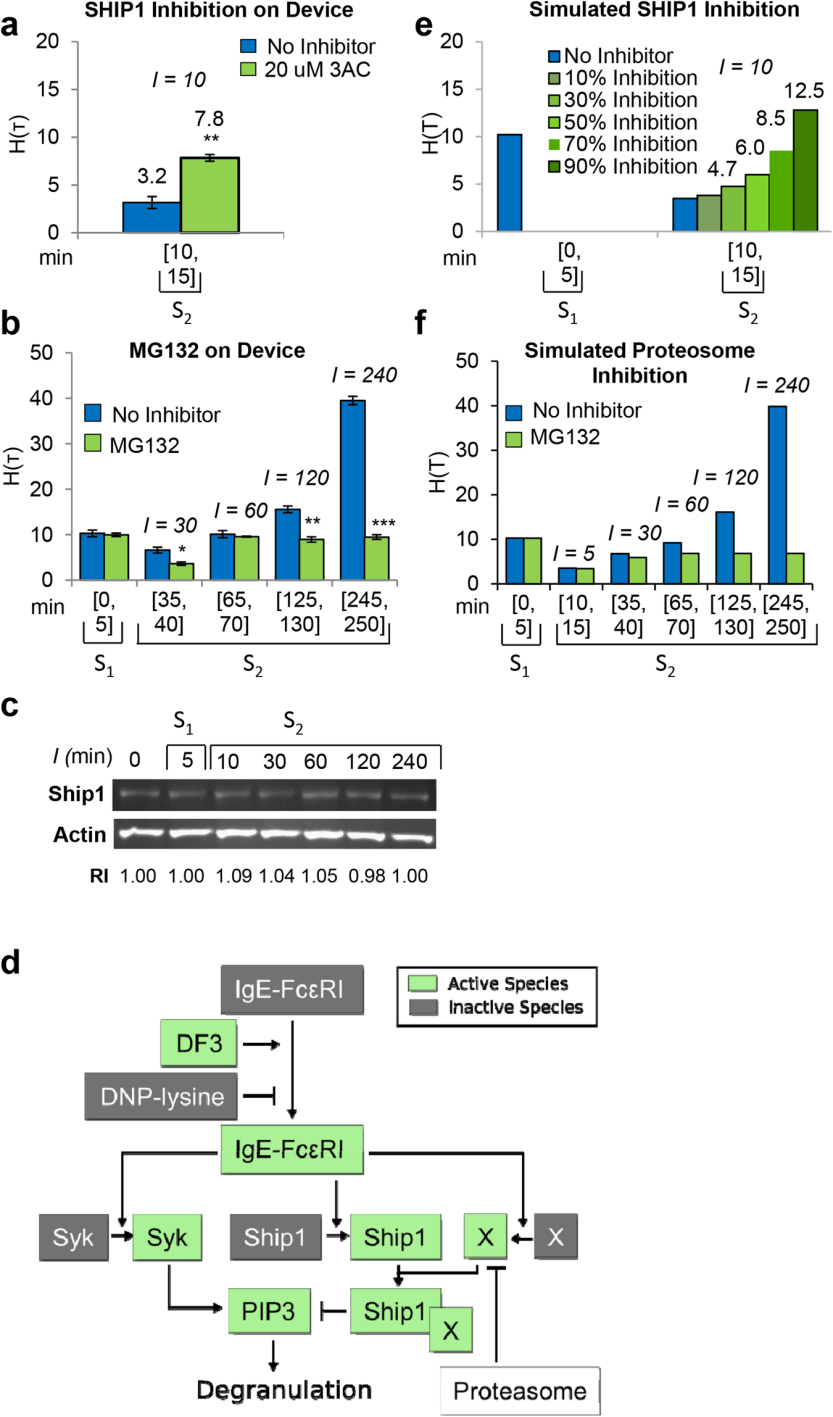
A mathematical model to explain the phenomena of desensitization and priming. (**a**) Degranulation in in the absence (no inhibitor, blue bars) or presence of Ship1 inhibitor (3AC, green bars), after pulsed stimulation in cell culture. Data are presented as mean ± S.D (n = 3), two-tailed paired *t*-test comparison of % H(_T_) values at S_2_ for 3AC treated versus untreated cells (**P < 0.01; *P* = 0.0029). (**b**) Degranulation in the absence (no inhibitor, blue bars) or presence of a proteasome inhibitor (MG132, green bars), after pulsed stimulation in cell culture; (**a,b**) Data are presented as mean ± S.D (n = 3), For each pair of untreated cells (blue bars) compared to treated cells (green bars), we performed a paired two-tailed *t*-test. Asterisks indicate statistically significant differences: ***P < 0.001; **P < 0.01; and *P < 0.05; *I* = 30 min, *P* = 0.021; *I* = 120 min, *P* = 0.0097; *I* = 240 min, *P* = 1.27 × 10^−4^). (**c**) Ship1 protein abundance over time measured by western blot. Data represent results from one of three experiments with similar results. **(d)** Diagram of the mathematical model. Activation of IgE-FcεRI by DF3 triggers activation of Syk, Ship1, and the Ship1 co-factor *X*. Syk and Ship1 exert opposing influences on degranulation. The proteasome degrades *X*. (**e)** Simulated degranulation in the absence (blue bars) or presence (green bars) of a Ship1 inhibitor. The inhibitor was simulated with several possible levels of efficacy, ranging from 10% to 90% inhibition of Ship1 activity. (**f)** Simulated degranulation in the absence (blue bars) or presence (green bars) of a proteasome inhibitor.

To understand the signaling dynamics that give rise to memory, we sought to perturb short- and long-term memory at the molecular level, and to incorporate this information into a model of the system that could reproduce experimental measurements of degranulation for a range of *I* values (Fig. 3). We first considered which negative regulators might bring about desensitization. The lipid phosphatase Ship1 is known to be important in downregulation of signaling by FcεRI^9^. To assess the role of Ship1 in desensitization, we treated cells during the *S*_2_ phase with a Ship1-specific inhibitor, 3-α-aminocholestane (3AC)^19^. To avoid dose-dependent 3AC cytotoxicity (Supplementary Fig. S7), we used a concentration of 20 µM. We found that the second secretory response of 7.83 ± 0.35% in the presence of 3AC was much greater than the *H(S*_2_) value of 3.16 ± 0.62% for untreated cells (Fig. 3a). These results indicate that Ship1 plays a role in desensitization. We also evaluated the effect of compound NSC-87877, an inhibitor of the tyrosine phosphatase Shp1, a protein that has been shown to positively and negatively regulate mast cell degranulation^9^. In contrast with inhibition of Ship1, inhibition of Shp1 did not affect desensitization (Supplementary Fig. S8).

We then considered what processes could counterbalance the effect of desensitization to bring about long-term memory. We hypothesized that protein degradation could be involved. To test this hypothesis, we treated cells with the proteasome inhibitor MG132. With MG132 treatment, we found that long-term memory was impaired when *I* was long (e.g., *I* = 240 min) (Fig. 3b). For *I* = 240 min, the value of *H(S*_2_ *)*, 9.46 ± 0.51%, was similar to that of *H(S*_1_ *)*, 9.97 ± 0.38%, for MG132-treated cells. In contrast, untreated cells were characterized by a value of *H(S*_2_), 39.5 ± 0.92%, that greatly exceeds that of *H(S*_1_), 9.78 ± 1.09% (Fig. 3b). Thus, the proteasome acts to inhibit the desensitization induced by Ship1.

We assessed whether these two players, Ship1 and the proteasome, are directly linked; that is, whether Ship1 is degraded in an inducible fashion by the proteasome. Surprisingly, we found that Ship1 was not degraded following antigen stimulation (Fig. 3c), indicating that the proteasome counteracts Ship1 activity in an indirect way. A possible mechanism of indirect negative regulation is proteasome-mediated degradation of one or more activators or co-factors of Ship1.

### A Computational Model Predicts Signaling Dynamics

**Figure 4:**
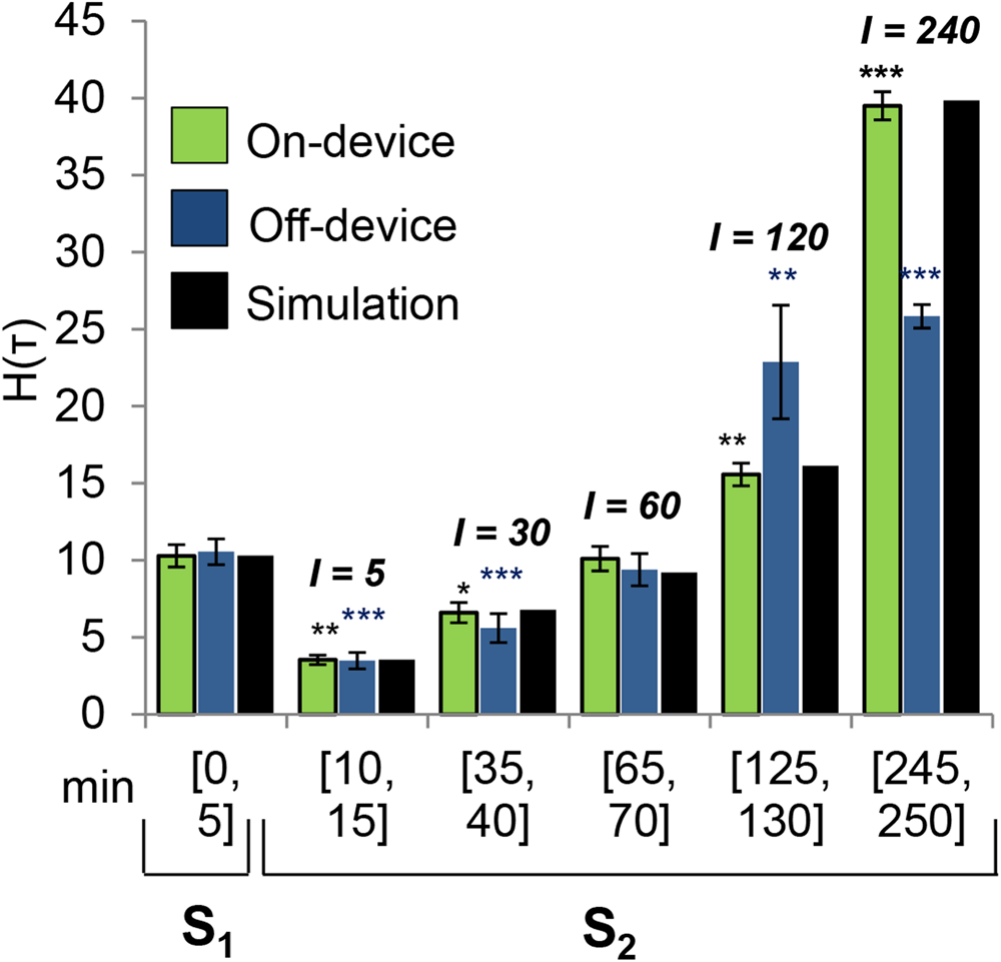
Simulated degranulation (black bars) vs. measured degranulation for cells cultured in the channel of the microfluidic device (green bars) or in 24-well plates (blue bars). The leftmost set of bars report *H(S*_1_). The other bars report *H*(*S*_2_) for *I* = 5, 30, 60, 120, or 240 min. *S*_1_ represents the initial 5 min stimulation with DF3, *S*_2_ is a second 5 min stimulation with DF3 after time period of signaling quiescence (DNP-lysine treatment) indicated by *I*. Shown are the means for three independent experiments performed in triplicate ± SD, paired two-tailed t test comparison of % *H*(_*T*_) values at *S*_2_ versus *S*_1_ (***P < 0.001; **P < 0.01; and *P < 0.05, on device: *I* = 10 min *P* = 0.006; *I* = 30 min, *P* = 0.044; *I* = 120 min, *P* = 0.0019; *I* = 240 min, *P* = 8.13 × 10^−4^. Off device: *I* = 10 min *P* = 5.98 × 10^−5^; *I* = 30 min, *P* = 1.67 × 10^−4^.; *I* = 60 min, *P* = 0.0038; *I* = 120 min, *P* = 3.07 × 10^−4^; *I* = 240 min, *P* = 0.0073.

To investigate the mechanisms controlling desensitization and priming, we used a rule-based approach^20,21^ to construct a mathematical model for the FcεRI signaling network consistent with known mechanisms of FcεRI signaling, principles of chemical kinetics, and the experimental clues that we had generated so far: 1) Ship1 negatively regulates signaling and 2) the proteasome negatively but indirectly regulates Ship1. In formulating the model, we hypothesized that a co-factor of Ship1, which we termed protein *X*, is degraded by the proteasome. The model is illustrated in Fig. 3d and described in detail in the Supplementary Note online. An executable, electronic version of the model is included in the online supplementary material (Supplementary Data S1); this file can be processed by the BioNetGen software package to produce simulation results^22,23^. Model parameter values were set, via fitting through use of the BioNetFit software package^24^, to reproduce the on-device data in Fig. 4, which were generated using the microfluidic device. The files used in the fitting procedure are included in the online supplementary material (Supplementary Data S2). The on-device data were validated in experiments using standard cell culture, washes, and media changes (Fig. 4, off-device). We observed a greater increase in degranulation at *I* = 240 in on-device experiments (*H(S*_2_), 39.5 ± 0.92%) vs. off-device experiments (*H(S*_2_), 25.8 ± 0.76%). However, trends were very similar. In both off-device and on-device experiments, there was a significant decrease in degranulation relative to the first response at shorter intervals of quiescence (*I* = 10 and *I* = 30 min) and a greatly enhanced second response at longer intervals of quiescence (*I* = 120 and *I* = 240 min).

After fitting, to test the ability of the calibrated model to make accurate predictions, we used the model to simulate degranulation responses in the presence and absence of Ship1 inhibition (Fig. 3e) and in the presence and absence of proteasome inhibition (Fig. 3f). The comparable experimental observations of system behavior for conditions with and without Ship1 inhibitor (3AC) and with and without proteasome inhibitor (MG132) are shown in Fig. 3a and b. It should be noted that these data were not used in fitting. As can be seen by comparing Fig. 3a and b with Fig. 3e and f, respectively, predictions are largely consistent with observations. As noted earlier, a 3AC concentration of 20 µM was used because higher concentrations were toxic to the cells (Supplementary Fig. S7). This concentration is perhaps suboptimal for inhibition of Ship1 activity, because according to our model, the observed level of degranulation in the presence of 20 µM 3AC, *H(S*_2_) = 7.83 ± 0.35%, is consistent with a 50–70% decrease in Ship1 activity (Fig. 3e). A discrepancy between predictions and observations is that the model predicts that MG132 should have essentially no effect on degranulation for an interval of signaling quiescence *I* equal to 30 min (Fig. 3f). In contrast, for the + MG132 condition relative to the –MG132 condition, we see about 2-fold less degranulation in experiments when *I* = 30 min (Fig. 3b). This finding suggests that proteosomal degradation is restraining degranulation on the minute to hour time scale after stimulation of IgE receptor signaling through a mechanism that is not captured in our model.

We used a Bayesian approach to quantify the uncertainties of parameter estimates and model predictions (Supplementary Note). Through this approach, we found that parameter estimates and predictions are reasonably robust.

### Syk and Ship1 are Activated and Deactivated on Different Timescales

**Figure 5:**
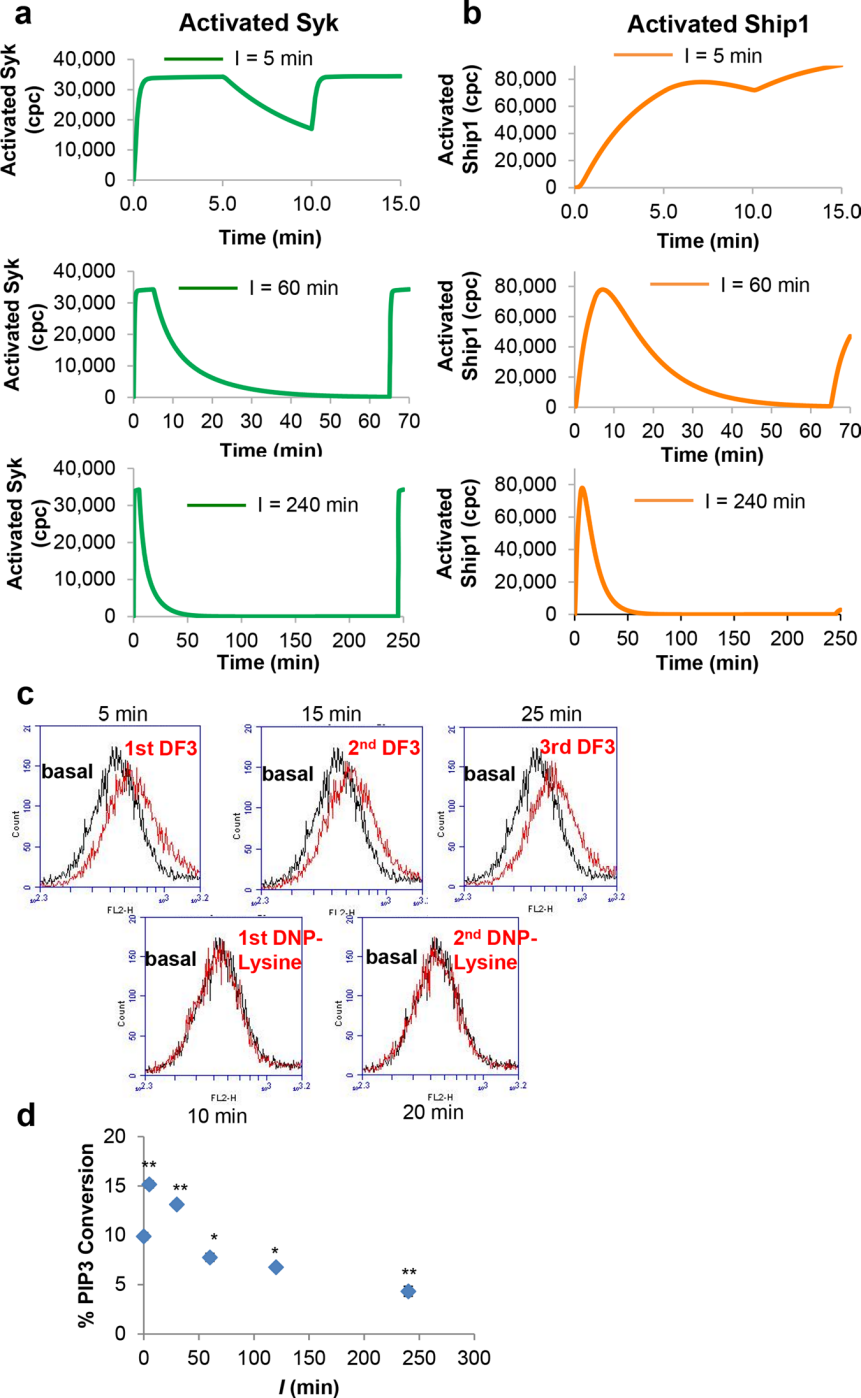
Simulated and measured dynamics of Syk and Ship1 activation/deactivation. (**a)** Simulation of copies per cell (cpc) of activated Syk (green line) for varying *I* values. (**b)** Simulation of cpc of activated Ship1 (orange line) for varying *I* values. **c)** Histograms of phosphorylation of Syk pY346, a readout of activation, as measured by flow cytometry during the same stimulation pattern as panel A (top). Cells were exposed to a 5-min pulse of DF3 (top left), followed by a 5-min pulse of DNP-lysine (10 min, bottom left), and then a second pulse of DF3 (15 min, top middle), followed by a 5-min pulse of DNP-lysine (20 min, bottom right), and then a third pulse of DF3 (25 min, top right). Measurements were taken for non-stimulated cells (basal) and at the end of each of these periods (red histograms); data are representative of results from three similar experiments. (**d)** Activation of Ship1 upon DF3 stimulation following the indicated *I* values, as measured by a malachite green assay with immunoprecipitated Ship1. Data are presented as mean ± SD (n = 3), *t*-test comparison of % PIP3 conversion values at *S*_1_ versus *S*_2_ (**P < 0.01; *P < 0.05; *I* = 5 min* P* = 0.0041; *I* = 30 min, *P* = 0.0045; *I* = 60 min, *P* = 0.0089; *I* = 120 min, *P* = 4.52 × 10^−4^; *I* = 240 min, *P* = 0.0055).

Through simulation-based analysis of the model, we found that the ability of the model to reproduce the experimental data depends on the following assumptions, which are incorporated in the model: 1) Syk is activated and deactivated quickly, 2) Ship1 is activated and deactivated slowly, and 3) the hypothesized co-factor of Ship1, *X*, is degraded very slowly. Our model predicts that the tyrosine kinase Syk, a known driver of positive signaling, undergoes rapid activation upon exposure to DF3, and rapid deactivation upon exposure to DNP-lysine. Rapid Syk activation and deactivation kinetics had been hypothesized previously but without direct experimental confirmation^25^. Our model also predicts that activation of Syk in response to a second DF3 pulse is largely history-independent, meaning that Syk is expected to be reactivated in a similar manner regardless of the length of *I* (Fig. 5a). In contrast, our model predicts a slower and history-sensitive regulation pattern for Ship1 (Fig. 5b). In the model, Ship1 activation and deactivation is more gradual, such that a second DF3 pulse after *I* = 5 min results in greater Ship1 activation during *S*_2_, because Ship1 retains a “head start”. However, over time Ship1 gradually loses its capacity to be activated due to degradation of the Ship1 co-factor *X*. With *I* = 60 min, Ship1 activation is similar to the initial pulse. With *I* = 240 min, Ship1 is refractory to activation due to lack of the hypothesized Ship1 co-factor (Fig. 5b). In our model, this loss of negative signaling is responsible for the hyperdegranulation occurring for longer *I* values.

To test these predictions, we assessed Syk regulation through measurement of Syk phosphorylation at Y346. Phosphorylation of this site relieves Syk auto-inhibition^17,18^. We found that phosphorylation of Y346 rapidly rises with DF3 stimulation and rapidly falls with DNP-lysine exposure as measured by flow cytometry (Fig. 5c), consistent with model predictions. We tested the prediction of slow Ship1 activation and deactivation kinetics using a Ship activity assay. As expected, the ability of Ship1 to dephosphorylate lipids becomes increasingly refractory to repeated stimulation with an increase in *I* (Fig. 5d). Phosphatase activities (% PIP3 conversion) at *I* = 5 min (15.16 ± 0.35%) and *I* = 30 min (13.13 ± 0.32%) are very similar; however, in comparison to activity at *I *= 5 min, there is a 49% decrease in activity at *I* = 60 min (7.76 ± 0.40%) and a 71% decrease in activity at *I* = 240 min (4.33 ± 0.50%).

### Shc1 Knockdown Accelerates Long-Term Memory

**Figure 6:**
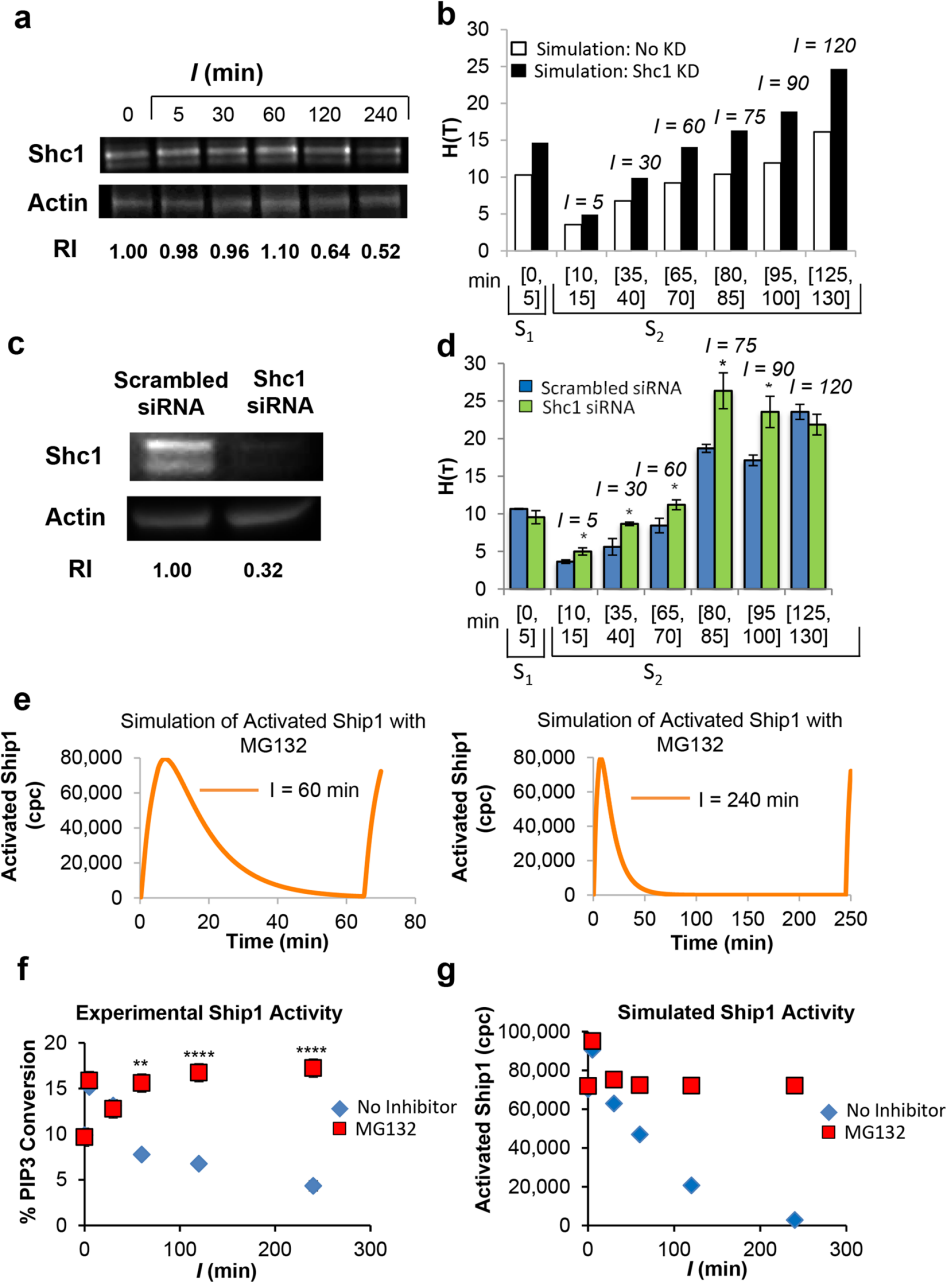
Role of Shc1 in memory responses. (**a)** Measured Shc1 abundance over time by western blot, the RI was calculated as the quotient of the densitometry signal for Shc1 band and that for actin and then normalized by the ratio obtained at time 0 (S_1_, considered 1). Data represent results from one of three experiments with similar results. (**b)** Predicted effect of Shc1 knockdown on memory (black bars), compare to simulations of WT cells without Shc knockdown (white bars). (**c)** Measured Shc1 protein expression by western blot in RBL-2H3 mast cells transfected with control scrambled siRNA or Rat Shc1 siRNA (Shc1 KD cells). Data represent results from one of three experiments with similar results. (**d)** Cell culture degranulation of WT (control scrambled siRNA, blue bars) and Shc1 KD cells (Rat Shc1 siRNA, green bars) for various *I* intervals; data are presented as mean ± S.D (n = 3), t test comparison of H(S_2_) values of control siRNA cells compared to that of Shc1 KD cells (*, P < 0.05; *I* = 15 min, *P* = 0.0497; *I* = 30 min, *P* = 0.026; *I* = 60 min, *P* = 0.037; *I* = 75 min, *P* = 0.039; *I* = 90 min, *P* = 0.0497). (**e)** Simulated time courses show the predicted effect of proteasome inhibition on Ship1 activation. The left panel shows dynamics of Ship1 activation for *I* = 60 min, and the right panel shows dynamics of activation for *I* = 240 min. Ship1 is robustly re-activated in both cases (**f)** Ship1 activation upon DF3 stimulation following the indicated *I* values, with proteasome inhibitor (MG132, red squares), or without inhibitor (no inhibitor, blue diamonds), as measured by a malachite green assay with immunoprecipitated Ship1. Data are presented as mean ± SD (n = 3), t test comparison of % PIP3 conversion values of untreated cells compared to that of MG132-treated cells (****P < 0.0001; ***P < 0.001; **P < 0.01; *I* = 5 min, *P* = 0.026; *I* = 60 min, *P* = 0.0058; *I* = 120 min, *P* = 1.12 × 10^−5^; *I* = 240 min, *P* = 8.01 × 10^−5^). (**g)** Simulated levels of Ship1 activity, measured as the number of copies of activated Ship1, in the presence (red squares) or absence (blue diamonds) of proteasome inhibition, at the same time points as shown in panel f.

Next, we sought to identify the hypothesized Ship1 co-factor (or co-factors) degraded by the proteasome. We considered a set of proteins that are known to interact with and contribute to Ship1 activity. The proteins that we were able to monitor by western blotting, such as Dok1 and Lyn, did not show changes in abundance following stimulation (Supplementary Fig. S9), with one exception. Shc1 underwent noticeable degradation over time, with a 36% decrease at 120 min and a 48% decrease in Shc1 expression at 240 min (Fig. 6a). Interestingly, Ship1 is recruited to a ternary complex with Shc1 and the adaptor LAT^26^, and Shc1 and Ship1 both interact with the β chain ITAM of FcεRI^27^. Shc1 is also part of a ternary complex with Ship1 and PKC-δ, another negative regulator of FcεRI-induced mast cell signaling^28^. For these reasons, we hypothesized that Shc1 may contribute to the priming and long-term memory phenomena.

To test this hypothesis, we considered the effect of a *Shc1* knockdown (KD). We first simulated the effect of the knockdown and found that hyper-degranulation is predicted to occur earlier (Fig. 6b). We then used siRNA to experimentally reduce the abundance of Shc1 in cells (Fig. 6c). Degranulation assays for wildtype (WT) and *Shc1* KD cells are consistent with predictions, with significant hyper-degranulation (*P* = 0.04), *H(S*_2_) equal to 24.16 ± 2.39%, arising earlier (*I* = 80 min) for *Shc1* KD cells than for WT cells [*I* = 80 min, *H(S*_2_) of 16.54 ± 0.90%; *I* = 90 min, *H(S*_2_) of 18.43 ± 1.95%; *I* = 120 min, *H(S*_2_) of 23.80 ± 0.52%] (Fig. 6d). The model also predicted that with proteasome inhibition, Ship1 activity would be elevated during pulse *S*_2_, for *I* = 60 min or 240 min (Fig. 6e). The qualitative shapes of the curves determined experimentally (Fig. 6f) and by simulation (Fig. 6g) match for the final level of Ship1 activity after *S*_2_, with or without proteasome inhibition. This concordance supports the model predictions shown in Fig. 6e. We conclude that Shc1 contributes to slowing the onset of mast cell hyperactivity (i.e., priming), presumably by supporting activation of Ship1.

## Discussion

A cell’s response to an environmental perturbation is determined by the internal dynamics of its information processing systems, which in turn can be influenced by what the cell has experienced in the past. Investigating the details of this relationship requires fine control of the cellular microenvironment, quantification of resultant changes in the biochemical state of a cell, and an understanding of the molecular network that connects inputs to outputs. For these purposes, microfluidic technology and computational modeling are two valuable tools, as illustrated here and in other recent studies^13,14^. We coupled the two to examine the internal dynamics of signaling induced by complex time-varying patterns of antigen stimulation of the high-affinity receptor for IgE (FcεRI). We found that mast cells exhibit varied responses depending on the interval of time between periods of stimulation: short intervals resulted in attenuated responses to a second antigen exposure (desensitization), whereas long intervals of input quiescence resulted in enhanced responses (priming).

In investigating the mechanisms underlying these contrasting responses, pulsed stimulation enabled us to consider both the kinetics of activation (upon exposure to multivalent DF3) and deactivation (upon exposure to monovalent DNP-lysine) on timescales of minutes to hours. We uncovered different timescales for activation and deactivation of signaling proteins. The protein tyrosine kinase Syk, which transmits signals that promote degranulation, undergoes rapid activation and deactivation in a history-independent manner. In contrast, the lipid phosphatase Ship1, which has a negative effect on degranulation^28^, undergoes slower activation and deactivation. The slower kinetics of Ship1 activation and deactivation gives rise to history-dependent responses. History dependence also arises from the much slower process of antigen-induced proteasome-mediated degradation of proteins, including Shc1, a binding partner of Ship1^26,29^, but not Ship1 itself. Although our data implicate Shc1 as a Ship1 cofactor, we cannot conclude that Shc1 is essential for the negative regulatory functions of Ship1 nor that degradation of Shc1 is primarily responsible for the loss of Ship1 activity that underlies the priming phenomenon. Indeed, our data suggest that other Ship1 cofactors, also targeted by the proteasome, are involved. As can be seen in Fig. 6a, there is no loss of Shc1 expression up to 60 min after initiation of IgE receptor signaling. Yet, Ship1 activity is suppressed when *I* = 60 min (Figs 5 d and 6f), and moreover, MG132 treatment relieves this suppression of Ship1 activity (Fig. 6f). These putative unknown cofactors of Ship1 that are more rapidly degraded than Shc1 are not accounted for in our model.

Priming is a feature of several types of immune cells. In a general sense, priming prepares a cell to respond robustly to antigen stimulation at a later time. Other immune cells, such as T and B cells, undergo priming when they encounter their specific antigens and begin differentiation. Mast cells can be primed by IL-4 or by pharmacological agents^30,31^. Here, we have shown that mast cells can also be primed by exposure to an antigen and that priming depends on proteosomal degradation. The possible biological relevance of this phenomenon is that prolonged exposure to antigens from, say, a parasite, which mast cells can defend an organism against^32^, may induce inflammatory responses that are amplified over time. Although our data indicate that the proteasome is responsible for priming at long times after initiation of IgE receptor signaling (hours), our data also suggest that the proteasome limits degranulation at shorter times (Fig. 3b). We have not investigated how the proteasome limits degranulation on the minute-to-hour time scale. We speculate that a Syk cofactor or an inhibitor of Ship1 may be subject to proteasome-mediated degradation immediately upon initiation of IgE receptor signaling.

Our results are consistent with known mechanisms of Syk and Ship1 activation, which involve recruitment of these proteins to the plasma membrane. Syk has a tandem pair of Src homology 2 (SH2) domains that each bind weakly to phosphorylated immunoreceptor tyrosine-based activation motifs (ITAMs) in the γ chains of FcεRI. Recent *in vivo* measurements indicate that the lifetime of Syk recruitment to the plasma membrane during IgE receptor signaling is ephemeral, on the order of seconds^33^. Furthermore, phosphorylation of Y130 (rat numbering), which is located near the tandem SH2 domains, can accelerate dissociation of Syk from ITAMs^33,34^.

In contrast, Ship1 activation may be more sustained and long lived. Ship1 is known to join multicomponent signaling complexes that are held together through multiple interfaces. For example, Ship1 forms a ternary complex with Shc1 and LAT^26^. Such complexes are potentially long-lived because a complex can break apart only after the sequential breakage of multiple bonds^35,36^. We caution that, given the number of proteins that interact with Ship1 and influence its recruitment to the plasma membrane and its enzymatic activity, Shc1 may not be the only relevant target of the proteasome or the sole factor responsible for priming and hyperactive degranulation responses to antigen stimulation. Additional signaling proteins subject to proteasome-mediated degradation may also contribute to the priming phenomenon.

The results presented here suggest a new function for Ship1 in IgE receptor signaling. This protein has already been characterized as a “gatekeeper” of mast cell signaling due to its decisive role in determining the magnitude of mast cell responses by regulating the abundances of important phospholipids^28^. Its presence also has a strong influence on the shape of a mast cell’s dose-response curve; in the absence of Ship1, degranulation shows a dramatic increase in magnitude across multiple antigen doses^28^. Our findings, characterizing its activation/deactivation dynamics and its role in determining frequency responses, reveal that it has a strong impact on determining how cells respond on long time scales (on the order of hours) to the duration and history of antigen stimulation.

The approach used here to uncover signaling dynamics in mast cells could be generalized to study the frequency response properties of other systems, e.g., how the frequency of stimuli impacts the magnitude of responses. Precisely timed addition and removal of a stimulus via microfluidic technology can potentially enable characterization of activation and deactivation dynamics in diverse cell signaling systems.

## Methods

### Cells and Reagents

RBL-2H3 cells were maintained in MEM (Invitrogen, Carlsbad, CA), with 10% FBS, 100 U/ml penicillin, and 100 U/ml streptomycin (Invitrogen) and l-glutamine at 37 °C under 5% CO_2_. Mouse monoclonal anti-DNP-IgE was prepared as described by Liu *et al*.^37^. Trivalent ligand DF3 and FAM-conjugated DF3 were custom synthesized by AnaSpec Inc. (Fremont, CA). DNP-lysine, 4-Methylumbelliferyl N-acetyl-β-d-glucosaminide (MUG) and phosphatase inhibitor were purchased from Sigma. RIPA lysis buffer and 10X Halt protease inhibitor were purchased from Thermo Scientific. Flexi Tube rat Shc1 siRNA (Rn_Shc1_1 FlexiTube siRNA) and control scrambled siRNA (AllStars negative-control siRNA), were purchased from Qiagen.

### Integrated Microfluidic Platform for Pulsed Stimulation

Polydimethylsiloxane (PDMS)-based microfluidic devices were fabricated using standard soft lithography. The PDMS channel was permanently sealed to No. 1 glass cover slips. One end of PEEK tubing (125 μm i.d.) was directly inserted into the PDMS to insure tight sealing, while the other end of the tubing was connected to the fast response electronic valve controller and active pressure controller (APC) to achieve precise control of fluidics within the channel. The temperature of the system was maintained by a thermostat attached to the chip manifold. A graphical user interface (GUI) was programmed to interface the rabbit control box with pressure control units and electronic valves.

Prior to on-chip assays, the assembled devices were placed in a desiccator under vacuum for 2 h to evacuate the PDMS bulk and prevent bubble formation within the channels in the course of cell-based assays. The devices were cleaned using 10% freshly prepared bleach solution, 70% ethanol and Hank’s buffered saline solution (HBSS). 10 μg/ml of human plasma fibronectin was used to enhance the attachment of cells to the glass surface. RBL-2H3 cells in suspension were injected into the device to achieve 5,000-10,000 cells in serpentine channels. After a 30 min static incubation of cells at 37 °C, cells were primed with 100 ng/ml of monoclonal anti-DNP IgE. Flow rates between 0.5 and 1.0 μL/min were used in the washing steps unless indicated otherwise.

A pulsed stimulation pattern over single cells within the channel was achieved by alternating trivalent ligand DF3 (for example, at 10 nM concentration for 5 min) and excess monovalent ligand DNP-lysine (for example, at 100 μM concentration for another 5 min). Downstream cell-based assays including microscopic imaging, flow cytometry analysis and degranulation assays were performed immediately before and after each DF3 pulse.

### Pulsed Stimulation in Cell Culture

RBL-2H3 cells were plated in 24-well plates and treated with 1 μg/ml of mouse monoclonal anti-DNP-IgE in complete medium overnight. Following overnight priming with IgE, cells were washed two times with Hanks Buffer to remove excess IgE, stimulated with 10 nM DF3 in Hanks Buffer (0.5 ml/well) for 5 min at 37 °C and 150 μl of sample was collected for the first 5 min time point *H(S*_1_). For all other time points the cells were washed 1X with Hanks buffer prior to addition of 100 μM of DNP-lysine in Hanks buffer for 5 min at 37 °C. The cells were washed 1X with Hanks buffer to remove DNP-lysine then complete medium was added for the indicated time intervals (*I*) (e.g., 5 min, 30 min, 90 min, 120 min, and 240 min). Each interval included an initial 5-min period of incubation with DNP-lysine. At these times, medium was removed and cells were re-stimulated with 10 nM DF3 for 5 min at 37 °C, then 150 μL of supernatant was collected in microcentrifuge tubes (*H(S*_2_)). After supernatants were collected 0.5 ml/well of 1% Triton in Hanks buffer (TX-100) was added to cells to release total β-hexosaminidase from the cells. Then 150 μl of cell lysate was collected in microcentrifuge tubes.

### Degranulation Assay

We monitored degranulation of live cells adherent in the channel of the microfluidic device and in tissue culture by assaying enzymatic activity of secreted β-hexosaminidase. The assay used is a modification of a previously reported protocol^15,37^. Briefly, 10 μL of effluent from the microfluidic device, or supernatant from a tissue culture well, was added to 10 μL of MUG (1 mM) in sodium acetate (pH 5) in 1 well of a black bottom 96-well plate. After 30 min incubation at 37 °C, 200 mM glycine (pH 10) buffer was used to stop the reaction. Fluorescence was measured using an excitation wavelength of 360 nm and emission wavelength of 450 nm. Degranulation was quantified as the amount of secreted β-hexosaminidase activity divided by the total β-hexosaminidase activity in whole-cell lysates times 100%. The background level of β-hexosaminidase activity due to spontaneous secretion was subtracted from each sample. Experiments were performed in duplicate or triplicate three or more times and the mean and standard deviation (SD) for each experiment were calculated.

### Transfection with siRNA

Rat Shc1 siRNA or scrambled control siRNA (Qiagen) were transfected into RBL-2H3 cells at a final concentration of 20 nM using Lipofectamine RNAiMAX (Invitrogen) according to the manufacturer’s instructions. Cells at 6-hours post-transfection were plated in 24-well plates and treated with 1 μg/ml of mouse monoclonal anti-DNP-IgE in complete medium overnight. Following 18 hour priming with IgE, cells were analyzed for degranulation upon DF3 stimulation (24 hours post-transfection). Knockdown with Shc1 siRNA is reported relative to knockdown with scrambled siRNA.

### Flow Cytometry Analysis of pSyk

Following stimulation, cells were fixed (4% Paraformaldehyde (PFA)) at desired time points and permeabilized with 0.2% Tween 20 for an additional 10 min. The cells were stained with PE-conjugated phospho-ZAP-70/Syk antibody (1:100) in Pharmingen staining buffer. Stained cells were detached from the channel surface after immersion in 1% trypsin-EDTA at 37 °C for 5 min using a high-pressure flow (4 to 5 psi). Detached cells were collected, re-suspended in PBS buffer and analyzed using an Accuri flow cytometer (BD Biosciences).

### Internalization

Internalization of IgE-FcεRI complexes was measured based on established protocols using acid stripping and flow cytometry. Briefly, cells were sensitized with IgE-Alexa^647^ and stimulated with pulsed DF3 ligand. Then, the cells were briefly washed with a stripping buffer (0.5 M of NaCl and 0.2 M acetic acid) at 4 °C for 5 min to remove the surface IgE from cells. For quantification, identical samples were processed for flow cytometry, with and without acid stripping.

### Inhibitor Treatment

RBL-2H3 cells were plated 24 hours before inhibitor treatment. The following inhibitors were resuspended in dimethyl sulfoxide (DMSO) then diluted in MEM medium to obtain the final concentrations used in experiments: SHP1/2 PTPase inhibitor NSC-87877 (1 μM) and proteasome inhibitor MG132 (10 μM). The Ship1 inhibitor 3AC was resuspended in 100% ethanol then diluted in MEM medium to obtain the final concentrations used in experiments. As a control, cells were also incubated with 50 μM DMSO or 8 μl of ethanol, representing the highest concentration of DMSO/ethanol to which the cells were exposed with the inhibitors listed above. Cells were pre-incubated with MEM, DMSO, ethanol, 3-α-aminocholestane (3AC, Ship1 inhibitor), NSC-87877 (NSC, Shp1 inhibitor), or MG132 (proteasome inhibitor) for 1–3 hours at 37 °C. Following inhibitor treatment, cells were evaluated in immunoblotting, cell viability assay and degranulation assay experiments as described elsewhere in this report.

To test the viability of cells treated with chemical inhibitors for 3 hours, PrestoBlue cell viability reagent (Life technologies) was added to treated cells in MEM medium to make a 1X final concentration, and cells were incubated for 30 min at 37 °C. Fluorescence was measured using an excitation wavelength of 560 nm and emission wavelength of 590 nm. The percent cell viability was determined by dividing the average of the inhibitor treated samples by the average of samples treated with ethanol alone, taking the control (ethanol-treated) cells as 100%.

### Western Blot Analysis of Protein/Inositol Phosphatases

Total expression levels of protein/inositol phosphatases including Lyn, Dok1, Ship1, and Shc1 (Santa Cruz biotechnology) were measured upon pulsed stimulation. Lysis buffer supplemented with phosphatase inhibitor and protease inhibitor (1:100) was used to lyse cells. Protein concentrations were measured using Bradford assay (Bio-Rad) and samples were analyzed by Western blot as previously described^38^ with primary antibodies from Santa Cruz biotechnology. For detection of Shc1 knockdown by western blot, rabbit anti-Shc1 primary antibody from BD Biosciences and Pierce HRP-conjugated polyclonal goat anti-rabbit secondary antibody were used. All blots were re-probed with rabbit anti-actin polyclonal antibody (Novus) and visualized with an Alpha Innotech (San Leandro, CA) imager using a Super- Signal West Femto sensitivity substrate (Thermo Scientific). The relative intensity (RI) was calculated as the quotient of the densitometry signal for the target protein band divided by that for actin, then normalized by the ratio obtained for the control sample.

### Biochemical Analysis of Ship1 Activity After Membrane Extraction

Membrane-associated Ship1 was isolated using the Mem-Per Plus Membrane Protein Extraction kit (Pierce), and Ship-specific phosphatase activity was measured using previously described methods^39^. Briefly, after stimulation with pulsed DF3, cells were permeabilized with mild detergent to release cytosolic proteins followed by a second detergent to solubilize membrane proteins. Ship1 was pulled down from the membrane fraction using a Ship1-specific antibody and Protein A agarose beads (Thermo Fisher Scientific). Beads were washed three times with TBS + 10 mM DTT. Isolated Ship1 was analyzed using a Malachite Green phosphatase assay kit (Echelon Biosciences). Ship1 was incubated with water-soluble substrate, phosphatidylinositol (3,4,5)-trisphosphate (PIP3), for 60 min at 37 °C. Free phosphate generated from the Ship1 activity was determined by measuring absorbance at 640 nm in a microplate reader. Percent of PIP3 conversion was determined for each time point as [(free phosphate in reaction, pmol) − (background phosphate, pmol)] × 100%/3000 pmol. Free phosphate in background is the value of phosphate in “substrate-only PIP_3_” controls.

### Modeling and Simulation

We formulated a chemical kinetic model for IgE receptor signaling using the BioNetGen Language (BNGL)^40^. In the model, interactions and other processes are represented by formal rules^20,41^. The model was simulated deterministically using the capabilities of BioNetGen, meaning that the ordinary differential equations (ODEs) derived from the rules of the model were integrated numerically using CVODE^42^ with BioNetGen’s default settings for algorithmic parameters^40^. The model input file is provided as Supplementary Data S1. The model parameters were estimated through fitting using the BioNetFit software package^24^. The input files for BioNetFit are provided as Supplementary Data S2.

### Statistical Analysis

Relative levels of degranulation (defined as the amount of secreted β-hexosaminidase divided by the total β-hexosaminidase from whole cells) determined using the live cell degranulation assay and measured by fluorescence using an excitation wavelength of 360 nm and emission wavelength of 450 nm at *S*_1_ versus *S*_2_ were compared using a paired two-tailed *t*-test for each individual experiment. All experiments were performed three or more time with similar results. Degranulation is quantified as the percentage of total β-hexosaminidase released in TX-100 cell lysates. Values obtained with inhibitors suspended in DMSO or ethanol were compared to DMSO or ethanol-treated samples, respectively. For siRNA treated samples, values were compared to wells with scrambled siRNA on the same plate. For plate assays, untreated and DMSO/ethanol-treated controls, or scrambled siRNA, were included on every plate (in each independent experiment). Probability (P) values were considered significant when they were < 0.05 (*), < 0.01 (**), < 0.001 (***), or < 0.0001 (****).

## Electronic supplementary material

Supplementary Figures and Note

LaTeX Supplementary File

Supplementary Dataset 1
