# Supplementary material for: Timescale Separation of Positive and Negative Signaling Creates History-Dependent Responses to IgE Receptor Stimulation
Source: Sci Rep. 2017 Nov 14;7:15586. doi: 10.1038/s41598-017-15568-2 (PMC5686181; doi:10.1038/s41598-017-15568-2)
Supplement: Supplementary file 1 — Supplementary Figures and Note [file 41598_2017_15568_MOESM1_ESM.pdf]

Supplementary Figures

Timescale Separation of Positive and Negative Signaling Creates History-Dependent Responses to IgE Receptor Stimulation

Brooke Harmon [1 ϕ], Lily A. Chylek [1 §], Yanli Liu [1 ϕ], Eshan D. Mitra [ɘ], Avanika Mahajan [†], Edwin A. Saada [ϕ], Benjamin R. Schudel [ϕ], David A. Holowka [§], Barbara A. Baird [§], Bridget S. Wilson [†], William S. Hlavacek [ɘ *] and Anup K. Singh [ϕ *]

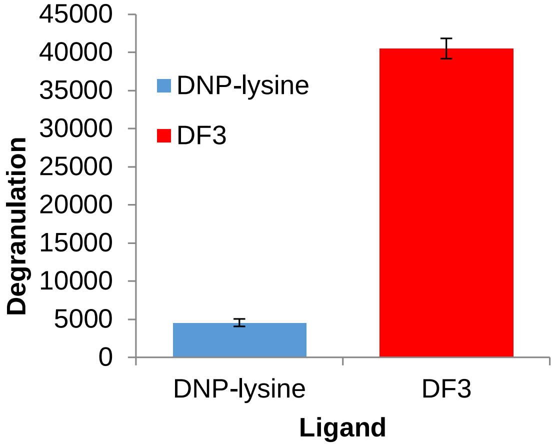

Supplementary Figure 1: DNP-lysine does not induce degranulation. Cells were first exposed to a 5-min pulse of DNP-lysine and then a 5-min pulse of DF3, and degranulation was measured during each of these periods. DNP-lysine did not induce appreciable degranulation.

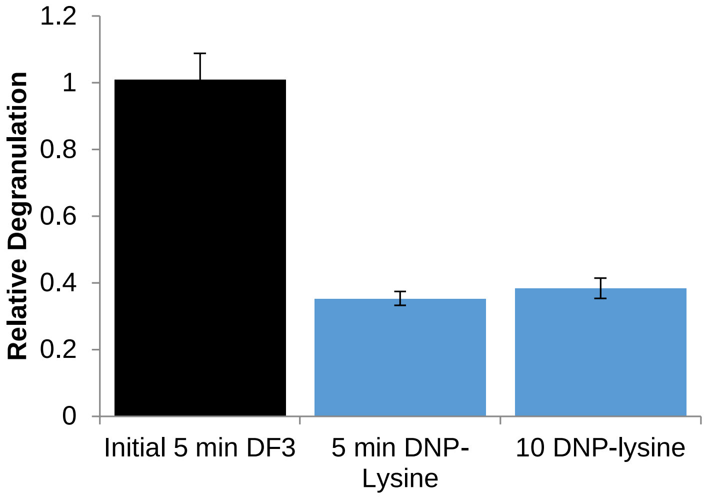

Supplementary Figure 2: 5 min of DNP-lysine is sufficient to break up receptor aggregates and reduce degranulation. Degranulation was monitored during an initial 5 min pulse of DF3, and then during a pulse of DNP-lysine that lasted either 5 or 10 min. Note that 5 min and 10 min of DNP-lysine exposure result in similar degranulation levels.

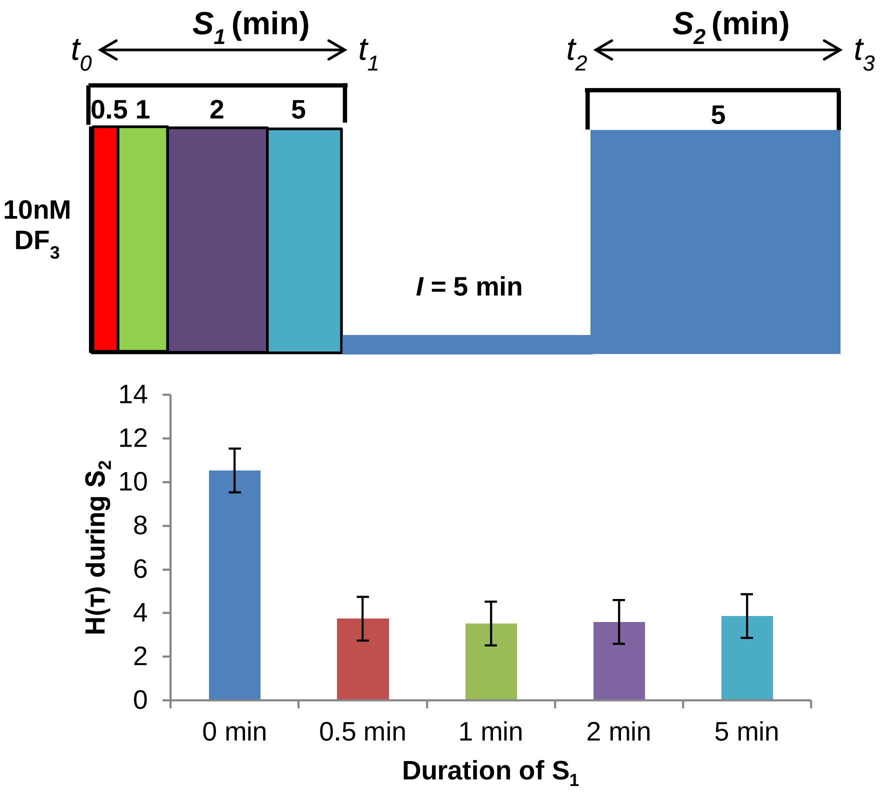

Supplementary Figure 3: The impact of S_1 duration on responses during S_2. The duration of S_1 was varied from 30 s to 5 min. The durations of I and S_2 were both 5 min in all cases. Desensitization occurs to a similar extent for all S_1 durations: the response during S_2 is alike for all of the S_1 durations considered.

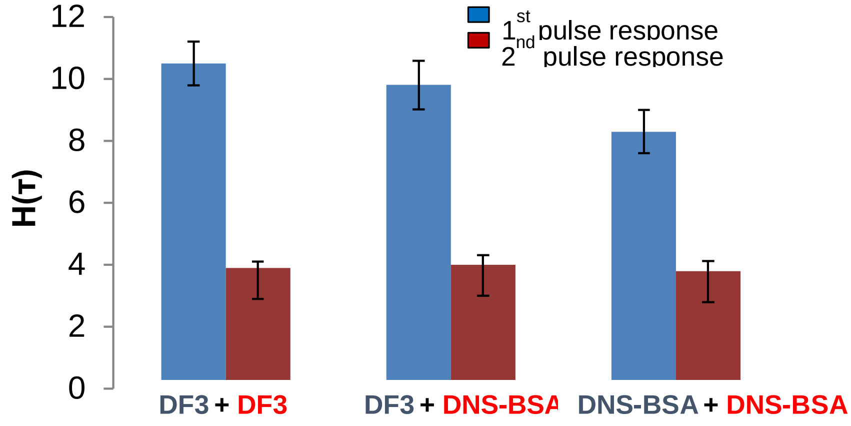

Supplementary Figure 4: Desensitization is nonspecific. Cells were sensitized with a half-and-half mixture of anti-DNP IgE, and anti-dansyl IgE. Cells were exposed to DF3 or a dansyl-BSA (DNS-BSA) in the indicated order. For all of these experiments, S_1 = 5 min, I = 5 min, and S_2 = 5 min. The blue bar represents the response to the first pulse and the red bar represents the response to the second pulse. Exposure to one antigen promotes desensitization to the other.

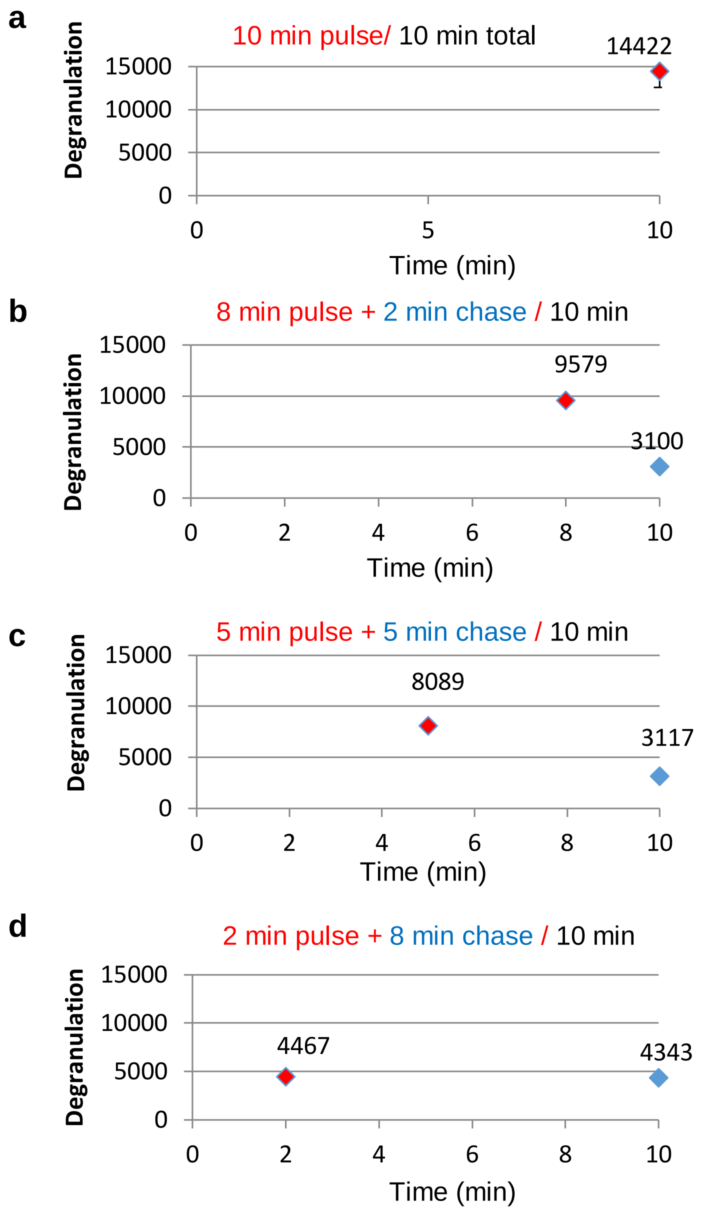

Supplementary Figure 5: Degranulation during continuous DF3 stimulation. A) cells were incubated with 10 nM DF3 for 10 min. B) Cells were incubated with 10 nM DF3 for the time shown in red (pulse), then DF3 was removed and cells were incubated for the time indicated in blue (chase) in media without DF3 for a total of 10 min. After each pulse with DF3 (10, 8, 5, or 2 min), supernatant was collected to measure degranulation prior to chase (red diamond, average degranulation values indicated above), and after the chase (blue diamond, average degranulation values indicated above). Degranulation increases for continuous stimulation up to 10 min. The increase in degranulation over this time period indicates that desensitization is not the result of a shortage of granules.

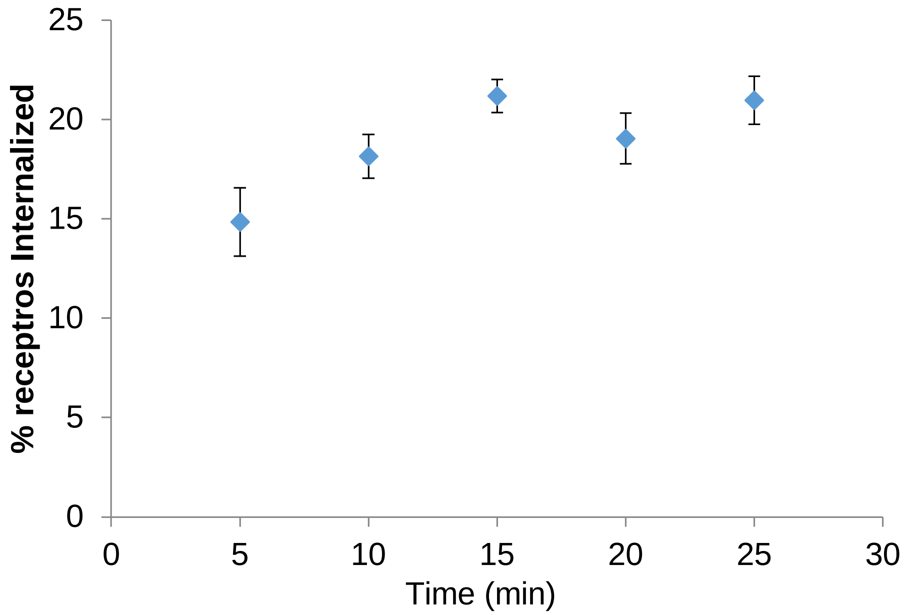

Supplementary Figure 6: Receptor internalization over time following DF3 stimulation

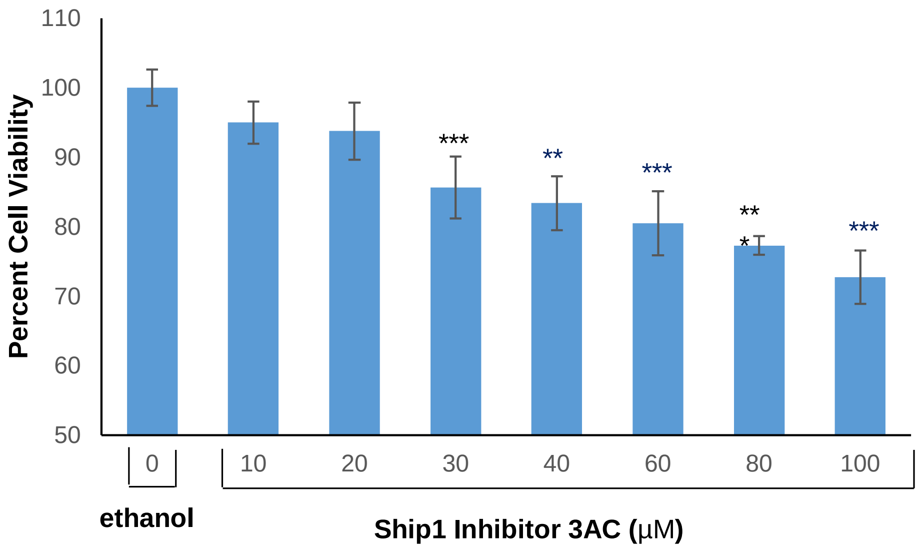

Supplementary Figure 7: RBL-2H3 cells were treated with ethanol alone (8 ul) or the indicated concentration of 3AC for 2 h. The percent cell viability was determined by taking ethanol-only treated cells as 100% (0 µM of 3AC). Three independent experiments were performed in triplicate. Data are presented as mean ±S.D. Asterisks indicate statistically significant differences: ***, P <0.001; **, P < 0.01; 30 µM, P = 0.0013; 40 µM, P = 0.004; 60 µM, P = $3.7X10^{-4}$; 80 µM P = $1.3X10^{-4}$; 100 µM, P = $3.6X10^{-4}$.

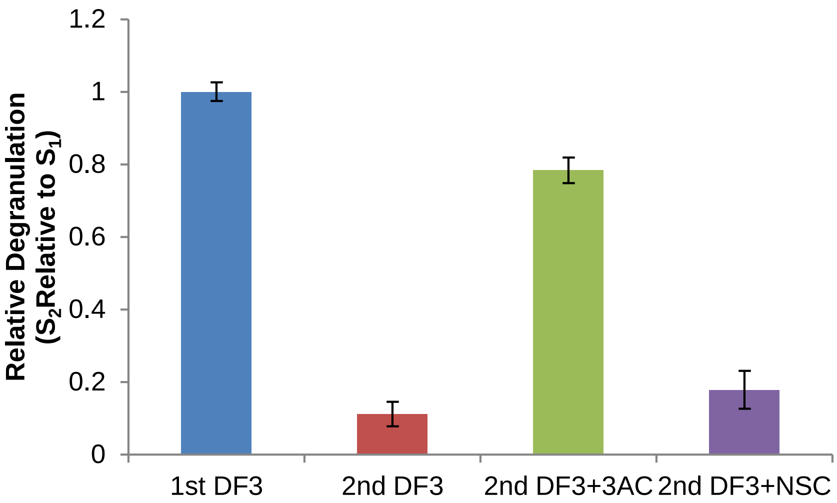

Supplementary Figure 8: Inhibition of the tyrosine phosphatase Shp1 (Ptpn6) has a minimal effect on desensitization. Cells were treated with 1 µM SHP1/2 PTPase inhibitor, NSC-87877 (NSC), or 20 µM SHIP1 inhibitor, 3AC, and then exposed to a stimulation pattern of S_1 = 5 min, *I* = 5 min, and S_2 = 5 min. The degranulation level of S_2 relative to that of S_1 for cells treated with 1 µM NSC, 0.18 ± 0.05, was similar to that of untreated cells (S_2 relative to S_1, 0.11 ± 0.03). In contrast, for cells treated with 3AC, there was clear desensitization with a much higher S_2 relative to S_1 (0.78 ± 0.04).

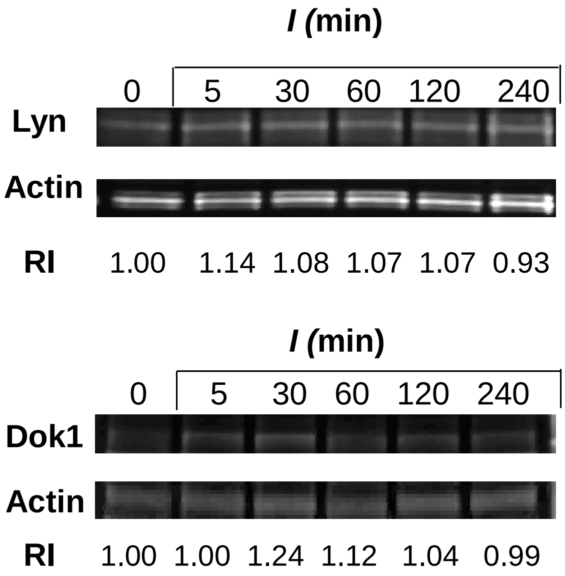

Supplementary Figure 9. Abundances of Lyn (top) and Dok1 (bottom) over time following stimulation with DF3. Both of these proteins are implicated in activation of Ship1, but their steady abundances do not agree with the model's predicted time course of degradation for protein X.

Supplementary Note

Timescale Separation of Positive and Negative Signaling Creates History-Dependent Responses to IgE Receptor Stimulation

Brooke Harmon, Lily A. Chylek, Yanli Liu, Eshan D. Mitra, Avanika Mahajan, Edwin A. Saada, Benjamin R. Schudel, David A. Holowka, Barbara A. Baird, Bridget S. Wilson, William S. Hlavacek and Anup K. Singh

Overview of the Model and Simulation Approach

We used the BioNetGen language (BNGL) to formulate a mathematical model for IgE receptor (FcϵRI) signaling with the goal of representing the opposing effects of Syk and Ship1 on antigen-stimulated secretory responses of antigen-sensitized mast cells. Syk is a protein tyrosine kinase recruited to activated FcϵRI that plays an important role in generating positive signals for mast cell degranulation, whereas Ship1 is a lipid phosphatase that plays an important role in generating negative signals. BNGL is a formal language for defining models (1; 2), which is akin to a domain-specific programming language. BNGL can be used to define not only models but also simulation protocols and simulation outputs. BNGL is compatible with the BioNetGen software package (1; 3), which includes various tools for deterministic and stochastic simulation of well-mixed chemical kinetics. In this study, we used the deterministic simulation capabilities of BioNetGen. The model is available as a plain-text BioNetGen input file named `model.bngl`. A listing of this file is provided in the Appendix. An electronic version is available as online supplementary material.

The file `model.bngl` consists of declarations that introduce 1) molecule types (e.g., formal representations of Syk and Ship1), 2) reaction rules and associated rate laws for molecular interactions and biochemical processes (e.g., a rule for recruitment of Syk to phosphorylated FcϵRI), 3) parameters (e.g., rate constants) and parameter settings, and 4) a list of seed species and their concentrations, which we took to define initial conditions for an initial value problem. The file `model.bngl` also includes a definition of simulation outputs, called observables, and simulation protocols/commands, called actions, such as `generate_network` and `simulate`.

Parameter fitting was performed using BioNetFit (4), a fitting program designed to work with BioNetGen. BioNetFit uses a genetic algorithm to stochastically find a best-fit parameter set for a model. BioNetFit was run on the plain text input file `model_fit.conf`, which configures BioNetFit with the appropriate model file (`model_tofit.bngl`) and experimental data files (`p1_5.exp`, `p3_5.exp`, `p3_30.exp`, `p3_60.exp`, `p1_120.exp`, `p1_240.exp`), as well as settings for fitting. As output from BioNetFit, we obtain a modified version of `model_tofit.bngl`, with the best-fit parameter values added to the beginning of the file.

The file `model.bngl` was processed by BioNetGen (1) to find the individual chemical species and reactions implied by the specified rules and seed species, as well as the corresponding set of coupled ordinary differential equations (ODEs) for well-mixed chemical kinetics. These ODEs were then integrated numerically. Numerical integration was performed by using CVODE in the SUNDIALS software package (5) with BioNetGen's default algorithmic settings, which are appropriate for stiff problems.

Below, we provide further information about the model and the computational methods used to analyze the model.

Molecule types

In the rule-based formulation of the model, we consider the following seven molecule types:

`Ag(DNP)`	#	antigen (DNP-conjugated BSA)
`R(IgE,Yb~0~P,Yg~0~P)`	#	antigen receptor (anti-DNP IgE-FcϵRI)
`Syk(tSH2)`	#	protein tyrosine kinase Syk
`Ship1(SH2,x)`	#	lipid phosphatase Ship1
`X(s~on~off)`	#	hypothetical Ship1 cofactor
`PIP3()`	#	phosphatidylinositol (3,4,5)-trisphosphate
`H(loc~in~out)`	#	hexosaminidase

The functional components of these molecules (i.e., their sites) are annotated in Table S1. Sites `Yb` and `Yg` in `R`, `s` in `X`, and `loc` in `H` are taken to have internal states. The internal states of `Yb` and `Yg` indicate if the sites are phosphorylated (`P`) or unmodified (`0`). The internal state of `s` indicates if `X` is competent (`on`) or incompetent for binding to Ship1 (`off`). The internal state of `loc` indicates the location of `H`: inside granules (`in`) or outside the cell

as a consequence of secretion (out). Pairs of cognate binding sites are identified in Table S2. These pairs of sites identify the four binary interactions considered in the model.

Table S1. The component sites of the molecule types considered in the model.

Site	Description
DNP in Ag	epitopes in DF3 recognized by anti-DNP IgE
IgE in R	antigen-combining sites in cell-surface anti-DNP IgE
Yb in R	ITAM tyrosines in the β subunit of FcϵRI, subject to phosphorylation.
Yg in R	ITAM tyrosines in the γ subunits of FcϵRI, subject to phosphorylation.
tSH2 in Syk	tandem SH2 domain structural subunit of Syk
SH2 in Ship1	SH2 domain in Ship1
x in Ship1	binding site in Ship1 responsible for interaction with X
s in X	binding site in X responsible for interaction with Ship1
loc in H	The state of this site indicates location: intra- or extracellular.

Table S2. Pairs of binding sites taken to interact with each other.

Binding site 1 (interaction partner of 2)	Binding site 2 (interaction partner of 1)
DNP in Ag	IgE in R
Yg with internal state P in R	tSH2 in Syk
Yb with internal state P in R	SH2 in SHIP
x in SHIP	s with internal state on in X

Rules

In the model, molecular interactions and other processes (e.g., proteasomal degradation of X) are represented by the following 14 rules, which are associated with 17 rate constants. For reversible rules, we define the forward rate constant, followed by the reverse rate constant.

Table S3. Reaction rules included in the model, and their rate constants.

Rule	Rate constants		Description
	in BNGL	in ODEs	
`Ag(DNP)+R(IgE)<-> Ag(DNP!1).R(IgE!1)`	`kon`, `koff`	k_{on}, k_{off}	Binding of ligand to receptor
`R(IgE!+,Yb~0,Yg~0)-> R(IgE!+,Yb~P,Yg~P)`	`kase`	k_{ase}	Phosphorylation of bound receptor
`R(Yb~P,Yg~P) ->R(Yb~0,Yg~0)`	`pase`	k_{pase}	Dephosphorylation of receptor
`R(Yg~P)+Syk(tSH2)<-> R(Yg~P!1).Syk(tSH2!1)`	`kp_Syk`, `km_Syk`	k_{pSyk}, k_{mSyk}	Recruitment of Syk to the γ subunit
`R(Yb~P)+Ship1(SH2)<-> R(Yb~P!1).Ship1(SH2!1)`	`kp_Ship1`, `km_Ship1`	k_{pShip}, k_{mShip}	Recruitment of Ship1 to the β subunit
`R(Yb~P!?)+X(s~off)-> R(Yb~P!?)+X(s~on)`	`k_Xon`	k_{Xon}	Activation of X by phosphorylated receptor
`X(s~on)->X(s~off)`	`k_Xoff`	k_{Xoff}	Deactivation of X
`Syk(tSH2!+)-> Syk(tSH2!+)+PIP3()`	`ksynth1`	k_{synth}	Syk-dependent synthesis of PIP3
`X(s~on)+Ship1(x)<-> X(s~on!1).Ship1(x!1)`	`kp_x`, `km_x`	k_{pX}, k_{mX}	Binding of active X to Ship1
`Ship1(SH2!+,x!+)+PIP3()-> Ship1(SH2!+,x!+)`	`kdeg1`	k_{deg}	Ship1-dependent dephosphorylation (clearance) of PIP3
`PIP3()->0`	`kpten`	k_{pten}	Basal clearance of PIP3
`X(s~on)->0`	`kdegX`	k_{degX}	Degradation of active free X by the proteasome
`X(s~on!1).Ship1(x!1)-> Ship1(x)`	`kdegX`	k_{degX}	Degradation of active Ship1-bound X by the proteasome
`PIP3()+H(loc~in)-> PIP3()+H(loc~out)`	`kdegran`	k_{degran}	Degranulation caused by the presence of PIP3

At this point, we should make note of the following simplifying assumptions. The receptor is taken to be monoclonal anti-DNP IgE in complex with FcϵRI, the high-affinity IgE receptor found on mast cells. The IgE-FcϵRI complex is long lived, on the order of hours to days. In the model, we represent the abundance of trivalent DNP ligand DF3 with an effective concentration of isolated DNP sites. When a receptor is not bound to a DNP site, we assume that it is unaggregated, and therefore, not competent to undergo receptor phosphorylation. In contrast, when a receptor is bound to a DNP site, we assume that it is aggregated and, accordingly, competent for phosphorylation. Receptor phosphorylation depends on receptor aggregation and is mediated predominantly by the protein tyrosine kinase Lyn, which we consider implicitly. We further assume that phosphorylation simultaneously occurs at the Ship1 binding site on the β chain and the Syk binding sites

on the γ chain. Finally, we assume that the concentration of PIP3 directly relates to the rate of degranulation. Downstream events, including the conversion of PIP3 to inositol trisphosphate (IP3), binding of IP3 to the IP3 receptor, and the subsequent mobilization of calcium, are considered implicitly.

Initial Conditions

Simulations began with initial quantities of each inactive molecule type (called seed species in BNGL). `R`, `Syk`, and `Ship1` were each initiated at 3e5 copies per cell. `H(loc~in)` had an initial count of 1e6. `PIP3` had an initial count of 0. The initial copy number of `X` was left as a free parameter, and was estimated through fitting to have a value of 1.84e6 copies per cell.

Simulation Protocol

Simulated time courses were run with conditions corresponding to experimental data. In the first phase of the simulation, the count of `Ag` was set to 3e6 copies, corresponding to a concentration of 10 nM in an extracellular volume of 0.5 nL per cell. This count of 3e6 was held constant even as `Ag` bound and unbound `R` (because of the approximately constant bulk concentration of antigen in the flow channel of the microfluidic device). The first phase of simulation was run for 5 min. The degranulation was reported as the final abundance of `H(loc~out)`, as a percentage of the total 1e6 copies of `H` per cell. In the second phase of simulation, `Ag` count was set to 0, to represent exchange of DF3 for excess DNP-lysine in the flow channel. This was run for a variable time interval of 5 to 240 min. Before the final phase of simulation, `H(loc~out)` was reset to 0, to measure only degranulation occurring in the final phase. Then `Ag` was reset to 3e6 copies, the simulation was run for another 5 min, and the final `H(loc~out)` count was recorded.

To simulate proteasome inhibition, we disabled the two rules corresponding to degradation of active free and Ship1-bound `X`. To simulate inhibition of Ship1 by 3AC, we multiplied the rate k_{deg} by a factor ranging from 0.1 to 0.9 (corresponding to varying possible levels of Ship1 inhibitor efficacy). To simulate a Shc1 knockdown, assuming `X` represents Shc1, we multiplied the initial concentration of `X` by 0.32, corresponding to the knockdown efficiency shown in Fig. 6C.

Parameters

We used BioNetFit to fit the 17 rate constants in Table S3, as well as the initial copy number of `X`. Parameters were fit to the experimental, on-device degranulation data shown in Fig. 4, which was tabulated in BioNetFit-readable format in the plain text files `p1_5.exp`, `p3_5.exp`, `p3_30.exp`, `p3_60.exp`, `p1_120.exp`, and `p1_240.exp`. Fitting was performed using a genetic algorithm with 300 generations, with 80 parameter sets per generation. The initial population of parameter sets was chosen according to a log uniform random distribution spanning 4 orders of magnitude around an initial guess for each parameter (see `model_fit.conf` for specific values). Simulation results for each parameter set were compared to the data with a chi-squared objective function.

In an initial fitting run, rate constants k_{on} and k_{deg} were set to values larger than a reasonable upper limit of 1e7 /M/s. These parameters were then set equal to 1e7 /M/s, and the fitting was repeated with the remaining 16 parameters free to vary.

The final fit parameters are given in the file `model.bngl`.

Bayesian Parameter Analysis

To assess our degree of uncertainty in the parameter values found with BioNetFit, we performed Bayesian Markov Chain Monte Carlo (MCMC) simulations to sample the credible region of parameter space. We followed the method described in (6). Briefly, for a parameter set Θ used to predict experimental data y, we seek to sample the posterior probability distribution $P(\Theta|y)$. By Bayes' theorem,

$$P(\Theta|y) = \frac{P(y|\Theta)P(\Theta)}{P(y)}$$

For the log likelihood $\ln P(y|\Theta)$, we use the negative of a χ^2 objective function, and for the prior $P(\Theta)$, we use a log (base 10) normal distribution, with mean equal to our value from BioNetFit and standard deviation of 1. $P(y)$ can be disregarded, as it remains constant for all Θ. Thus we can compute $P(\Theta|y)$ by evaluating the prior and objective function for any parameter set Θ.

To perform a step of the MCMC simulation, we propose a new parameter set Θ^* (by perturbing Θ by a distance of 0.1 in a random direction in log

(base 10) space), and compute $P(\Theta^*|y)$. The proposed move is accepted with probability $\min(1, P(\Theta^*|y)/P(\Theta|y))$. Our MCMC simulations consisted of 30 independent runs of 200000 steps each. Θ was recorded every 20 steps after an initial 20000-step burn-in period.

From this analysis, we obtain the marginal probability distribution of each parameter value (Fig. S10). This distribution indicates the range of reasonable values for each parameter; a narrower distribution indicates that the model is more sensitive to the value of that parameter.

Additionally, we assessed the robustness of the predictions of our model. For this analysis, we arbitrarily chose 540 parameter sets that were sampled during the MCMC simulation. We consider the predicted degranulation data, as shown in Fig. 4, for each of the sampled parameter sets. At each time point, the middle 68% of the predictions gives our 68% credible interval. In Fig. S11, we present this credible interval for each time interval tested. The credible interval forms a narrow band around each predicted value, which indicates that the degranulation predictions are quite consistent throughout the sampled parameter space.

Similarly, we evaluated the Syk and Ship1 activation time courses, as shown in Fig. 5a-b. At each time point, we considered the Syk or Ship1 activation level with each of our 540 sampled parameter sets, and took the middle 68% of those values to define the 68% credible interval at that time point. The credible intervals for the entire time courses are shown in Fig. S12. We find that these credible intervals preserve the qualitative features of the time course, indicating that this qualitative behavior is robust.

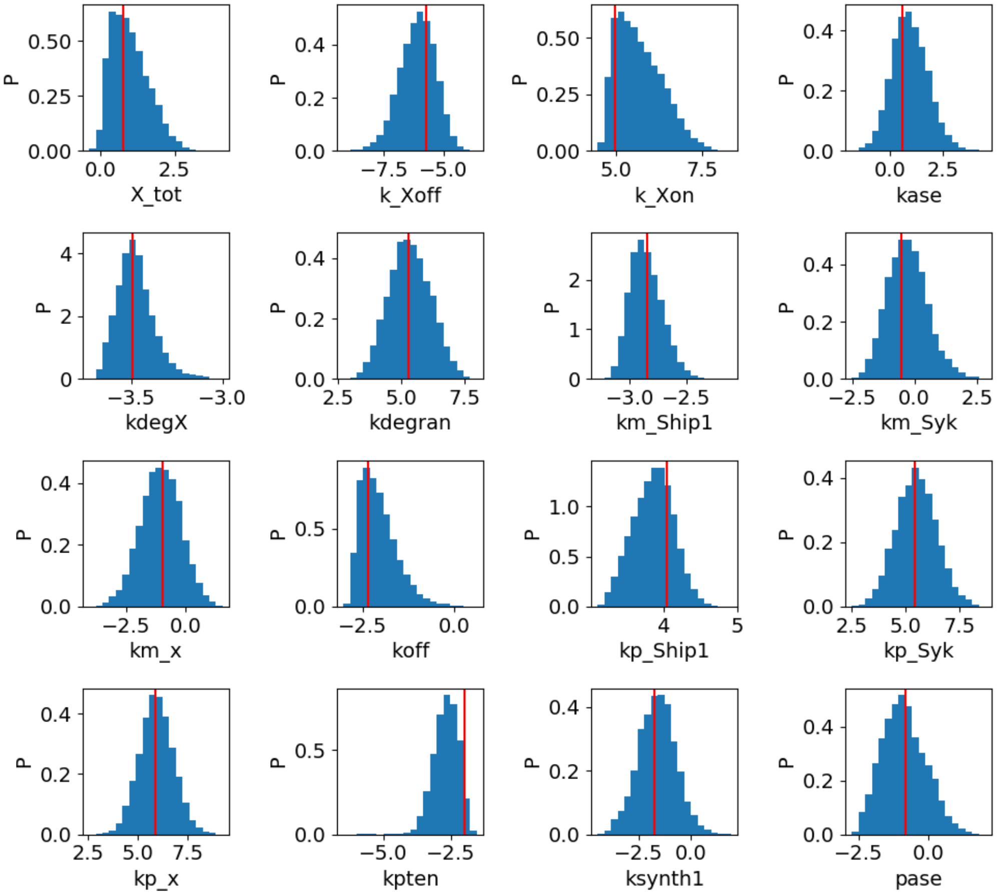

Figure S10: Marginal probability distributions for each parameter of the model. X-axes indicate the log base 10 of the parameter values. Red lines indicate the parameter value found by BioNetFit, which was used in the model for the rest of the study, and served as a prior for the Bayesian algorithm. Units are s^{-1} for first order rate constants, and $M^{-1}s^{-1}$ for second order rate constants. X_tot is expressed as the ratio of X concentration to Ship1 concentration.

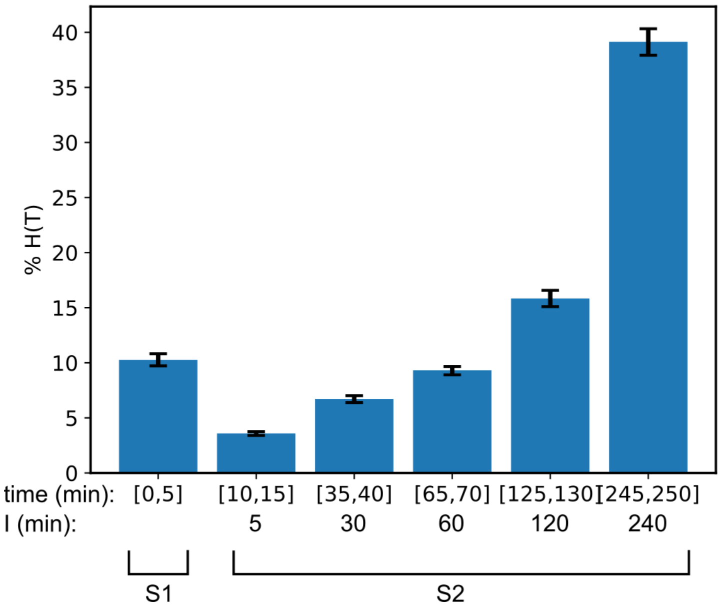

Figure S11: The robustness of the degranulation predictions of the model was characterized by Bayesian parameter estimation. Bar heights denote the median value of the Bayesian sample. Error bars denote the 68% credible region.

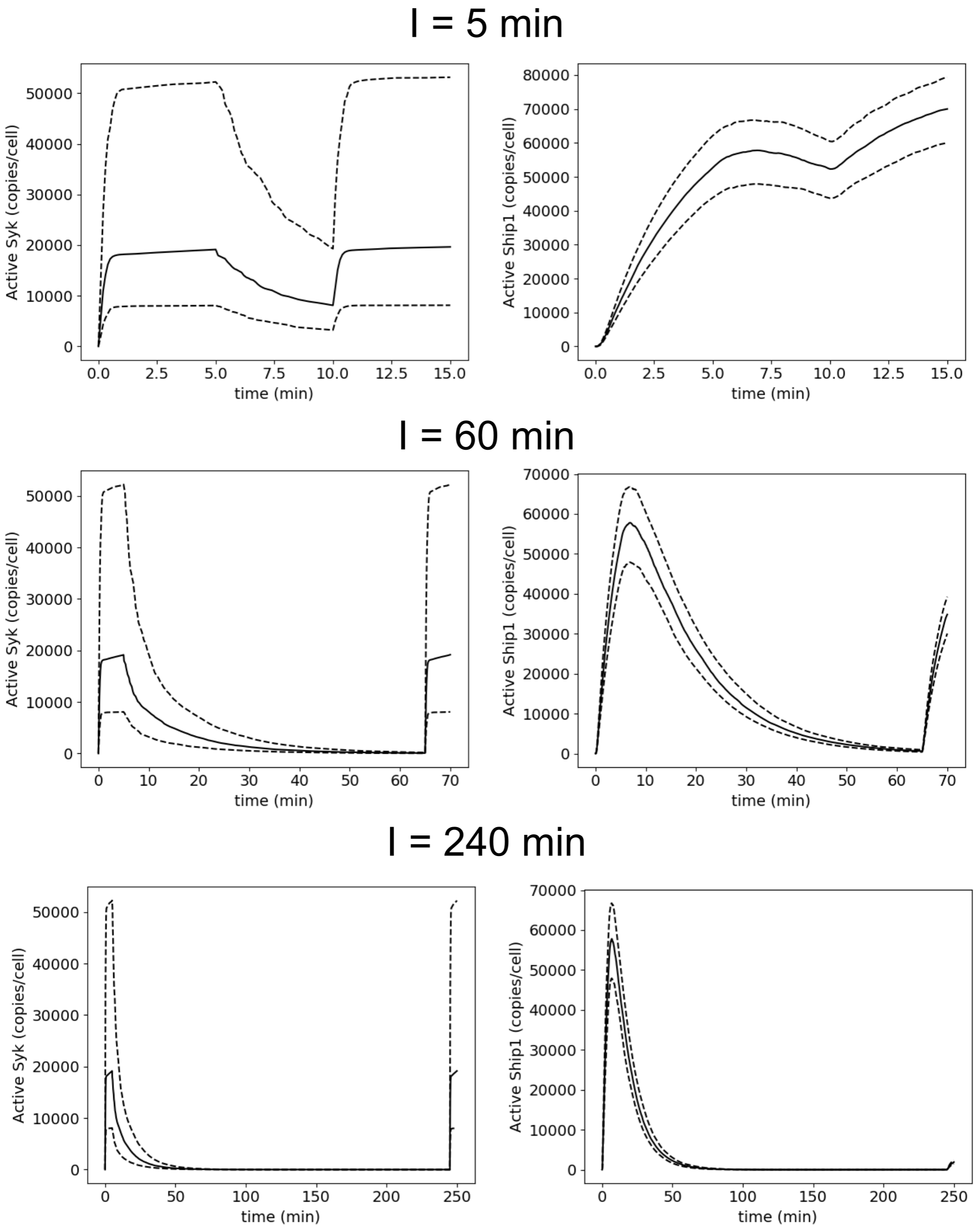

Figure S12: The robustness of Syk and Ship1 activation time courses was characterized by Bayesian parameter estimation. In each panel, dotted lines indicate the 68% credible region, and the solid line indicates the median of the Bayesian sample.

The chemical reaction network implied by rules and seed species

Table S4 lists the 23 individual chemical species that can be populated according to the rules of the model.

Table S4. Abbreviated names of chemical species.

Symbol	Representation in BNGL
x_1	`Ag(DNP)`
x_2	`R(IgE,Yb~0,Yg~0)`
x_3	`Syk(tSH2)`
x_4	`Ship1(SH2,x)`
x_5	`PIP3()`
x_6	`H(loc~in)`
x_7	`X(s~off)`
x_8	`Ag(DNP!1).R(IgE!1,Yb~0,Yg~0)`
x_9	`H(loc~out)`
x_{10}	`Ag(DNP!1).R(IgE!1,Yb~P,Yg~P)`
x_{11}	`R(IgE,Yb~P,Yg~P)`
x_{12}	`Ag(DNP!1).R(IgE!1,Yb~P,Yg~P!2).Syk(tSH2!2)`
x_{13}	`Ag(DNP!1).R(IgE!1,Yb~P!2,Yg~P).Ship1(SH2!2,x)`
x_{14}	`X(s~on)`
x_{15}	`R(IgE,Yb~P,Yg~P!1).Syk(tSH2!1)`
x_{16}	`R(IgE,Yb~P!1,Yg~P).Ship1(SH2!1,x)`
x_{17}	`Ag(DNP!1).R(IgE!1,Yb~P!2,Yg~P!3).Ship1(SH2!2,x).Syk(tSH2!3)`
x_{18}	`Ship1(SH2,x!1).X(s~on!1)`
x_{19}	`Ag(DNP!1).R(IgE!1,Yb~P!2,Yg~P).Ship1(SH2!2,x!3).X(s~on!3)`
x_{20}	`R(IgE,Yb~P!1,Yg~P!2).Ship1(SH2!1,x).Syk(tSH2!2)`
x_{21}	`R(IgE,Yb~P!1,Yg~P).Ship1(SH2!1,x!2).X(s~on!2)`
x_{22}	`Ag(DNP!1).R(IgE!1,Yb~P!2,Yg~P!3).Ship1(SH2!2,x!4).Syk(tSH2!3).X(s~on!4)`
x_{23}	`R(IgE,Yb~P!1,Yg~P!2).Ship1(SH2!1,x!3).Syk(tSH2!2).X(s~on!3)`

Network generation by BioNetGen yields the following list of 23 differential equations that define the model. These equations were numerically integrated to obtain simulation results.

$$
\begin{aligned}
\dot{x_1} &= -k_{on}x_1x_2 + k_{off}x_8 + k_{off}x_{10} - k_{on}x_1x_{11} \\
&\quad +k_{off}x_{12} + k_{off}x_{13} - k_{on}x_1x_{15} - k_{on}x_1x_{16} + k_{off}x_{17} \\
&\quad +k_{off}x_{19} - k_{on}x_1x_{20} - k_{on}x_1x_{21} - k_{on}x_1x_{23} + k_{off}x_{22} \\
\dot{x_2} &= -k_{on}x_1x_2 + k_{off}x_8 + k_{pase}x_{11} \\
\dot{x_3} &= -k_{pSyk}x_{10}x_3 - k_{pSyk}x_{11}x_3 - k_{pSyk}x_{13}x_3 + k_{mSyk}x_{12} \\
&\quad -k_{pSyk}x_{16}x_3 - k_{pSyk}x_{19}x_3 + k_{mSyk}x_{15} + k_{mSyk}x_{17} - k_{pSyk}x_{21}x_3
\end{aligned}
$$

$$
\begin{aligned}
& +k_{mSyk}x_{20} + k_{mSyk}x_{22} + k_{mSyk}x_{23} \\
\dot{x_4} &= -k_{pShip}x_{10}x_4 - k_{pShip}x_{11}x_4 - k_{pShip}x_{12}x_4 + k_{mShip}x_{13} \\
& -k_{pX}x_{14}x_4 - k_{pShip}x_{15}x_4 + k_{mShip}x_{16} + k_{mShip}x_{17} + k_{mX}x_{18} \\
& +k_{degX}x_{18} + k_{mShip}x_{20} \\
\dot{x_5} &= -k_{pten}x_5 + k_{synth}x_{12} + k_{synth}x_{15} + k_{synth}x_{17} \\
& -k_{deg}x_{19}x_5 + k_{synth}x_{20} + k_{synth}x_{22} + k_{synth}x_{23} - k_{deg}x_{21}x_5 \\
& -k_{deg}x_{22}x_5 - k_{deg}x_{23}x_5 \\
\dot{x_6} &= -k_{degran}x_5x_6 \\
\dot{x_7} &= -k_{Xon}x_{10}x_7 - k_{Xon}x_{11}x_7 - k_{Xon}x_{12}x_7 - k_{Xon}x_{13}x_7 \\
& +k_{Xoff}x_{14} - k_{Xon}x_{15}x_7 - k_{Xon}x_{16}x_7 - k_{Xon}x_{17}x_7 - k_{Xon}x_{19}x_7 \\
& -k_{Xon}x_{20}x_7 - k_{Xon}x_{21}x_7 - k_{Xon}x_{22}x_7 - k_{Xon}x_{23}x_7 \\
\dot{x_8} &= +k_{on}x_1x_2 - k_{off}x_8 - k_{ase}x_8 + k_{pase}x_{10} \\
\dot{x_9} &= +k_{degran}x_5x_6 \\
\dot{x_{10}} &= +k_{ase}x_8 - k_{off}x_{10} - k_{pase}x_{10} - k_{pSyk}x_{10}x_3 \\
& -k_{pShip}x_{10}x_4 + k_{on}x_1x_{11} + k_{mSyk}x_{12} + k_{mShip}x_{13} - k_{pShip}x_{10}x_{18} \\
& +k_{mShip}x_{19} \\
\dot{x_{11}} &= +k_{off}x_{10} - k_{on}x_1x_{11} - k_{pase}x_{11} - k_{pSyk}x_{11}x_3 \\
& -k_{pShip}x_{11}x_4 + k_{mSyk}x_{15} - k_{pShip}x_{11}x_{18} + k_{mShip}x_{16} + k_{mShip}x_{21} \\
\dot{x_{12}} &= +k_{pSyk}x_{10}x_3 - k_{off}x_{12} - k_{mSyk}x_{12} - k_{pShip}x_{12}x_4 \\
& +k_{on}x_1x_{15} - k_{pShip}x_{12}x_{18} + k_{mShip}x_{17} + k_{mShip}x_{22} \\
\dot{x_{13}} &= +k_{pShip}x_{10}x_4 - k_{off}x_{13} - k_{pSyk}x_{13}x_3 - k_{mShip}x_{13} \\
& -k_{pX}x_{14}x_{13} + k_{on}x_1x_{16} + k_{mSyk}x_{17} + k_{mX}x_{19} + k_{degX}x_{19} \\
\dot{x_{14}} &= +k_{Xon}x_{10}x_7 + k_{Xon}x_{11}x_7 + k_{Xon}x_{12}x_7 + k_{Xon}x_{13}x_7 \\
& -k_{Xoff}x_{14} - k_{pX}x_{14}x_4 - k_{pX}x_{14}x_{13} - k_{degX}x_{14} + k_{Xon}x_{15}x_7 \\
& +k_{Xon}x_{16}x_7 + k_{Xon}x_{17}x_7 + k_{Xon}x_{19}x_7 - k_{pX}x_{14}x_{16} - k_{pX}x_{14}x_{17} \\
& +k_{mX}x_{18} + k_{mX}x_{19} + k_{Xon}x_{20}x_7 + k_{Xon}x_{21}x_7 + k_{Xon}x_{22}x_7 \\
& +k_{Xon}x_{23}x_7 - k_{pX}x_{14}x_{20} + k_{mX}x_{21} + k_{mX}x_{22} + k_{mX}x_{23} \\
\dot{x_{15}} &= +k_{off}x_{12} + k_{pSyk}x_{11}x_3 - k_{on}x_1x_{15} - k_{mSyk}x_{15} \\
& -k_{pShip}x_{15}x_4 - k_{pShip}x_{15}x_{18} + k_{mShip}x_{20} + k_{mShip}x_{23} \\
\dot{x_{16}} &= +k_{off}x_{13} + k_{pShip}x_{11}x_4 - k_{on}x_1x_{16} - k_{pSyk}x_{16}x_3 \\
& -k_{mShip}x_{16} - k_{pX}x_{14}x_{16} + k_{mSyk}x_{20} + k_{mX}x_{21} + k_{degX}x_{21}
\end{aligned}
$$

$$
\begin{aligned}
\dot{x_{17}} &= +k_{pSyk}x_{13}x_3 + k_{pShip}x_{12}x_4 - k_{off}x_{17} - k_{mSyk}x_{17} \\
&\quad -k_{mShip}x_{17} - k_{pX}x_{14}x_{17} + k_{on}x_1x_{20} + k_{mX}x_{22} + k_{degX}x_{22} \\
\dot{x_{18}} &= +k_{pX}x_{14}x_4 - k_{pShip}x_{10}x_{18} - k_{pShip}x_{11}x_{18} - k_{pShip}x_{12}x_{18} \\
&\quad -k_{pShip}x_{15}x_{18} + k_{mShip}x_{19} - k_{mX}x_{18} - k_{degX}x_{18} + k_{mShip}x_{21} \\
&\quad +k_{mShip}x_{22} + k_{mShip}x_{23} \\
\dot{x_{19}} &= +k_{pX}x_{14}x_{13} - k_{off}x_{19} - k_{pSyk}x_{19}x_3 + k_{pShip}x_{10}x_{18} \\
&\quad -k_{mShip}x_{19} - k_{mX}x_{19} - k_{degX}x_{19} + k_{on}x_1x_{21} + k_{mSyk}x_{22} \\
\dot{x_{20}} &= +k_{off}x_{17} + k_{pSyk}x_{16}x_3 + k_{pShip}x_{15}x_4 - k_{on}x_1x_{20} \\
&\quad -k_{mSyk}x_{20} - k_{mShip}x_{20} - k_{pX}x_{14}x_{20} + k_{mX}x_{23} + k_{degX}x_{23} \\
\dot{x_{21}} &= +k_{off}x_{19} + k_{pShip}x_{11}x_{18} + k_{pX}x_{14}x_{16} - k_{on}x_1x_{21} \\
&\quad -k_{pSyk}x_{21}x_3 + k_{mSyk}x_{23} - k_{mShip}x_{21} - k_{mX}x_{21} - k_{degX}x_{21} \\
\dot{x_{22}} &= +k_{pSyk}x_{19}x_3 + k_{pShip}x_{12}x_{18} + k_{pX}x_{14}x_{17} + k_{on}x_1x_{23} \\
&\quad -k_{off}x_{22} - k_{mSyk}x_{22} - k_{mShip}x_{22} - k_{mX}x_{22} - k_{degX}x_{22} \\
\dot{x_{23}} &= +k_{pShip}x_{15}x_{18} - k_{on}x_1x_{23} + k_{off}x_{22} + k_{pSyk}x_{21}x_3 \\
&\quad -k_{mSyk}x_{23} - k_{mShip}x_{23} + k_{pX}x_{14}x_{20} - k_{mX}x_{23} - k_{degX}x_{23}
\end{aligned}
$$

References

[1] Faeder, J. R., Blinov, M. L. & Hlavacek, W. S. Rule-based modeling of biochemical systems with BioNetGen. *Methods Mol Biol* **500**, 113–167 (2009).

[2] Hogg, J. S., Harris, L. A., Stover, L. J., Nair, N. S. & Faeder, J. R. Exact hybrid particle/population simulation of rule-based models of biochemical systems. *PLoS computational biology* **10**, e1003544 (2014).

[3] Harris, L. A. *et al.* BioNetGen 2.2: advances in rule-based modeling. *Bioinformatics* **32**, 3366–3368 (2016).

[4] Thomas, B. R. *et al.* BioNetFit: a fitting tool compatible with BioNetGen, NFsim and distributed computing environments. *Bioinformatics* **32**, 798–800 (2016).

[5] Hindmarsh, A. C. *et al.* SUNDIALS: Suite of Nonlinear and Differential/Algebraic Equation Solvers. *ACM Trans. Math. Softw.* **31**, 363–396 (2005).

[6] Kozer, N. *et al.* Exploring higher-order EGFR oligomerisation and phosphorylationa combined experimental and theoretical approach. *Mol. BioSyst. Mol. BioSyst* **9**, 1849–1863 (2013).

Appendix

Listing of the BNGL model file

```
# filename:  model.bngl
# date of last edit:  25-Jul-2017
# authors:  Chylek LA, Mitra E, Hlavacek WS (Los Alamos National Laboratory)
# software compatibility:  RuleBender (version 2.1.0.8),
# BioNetGen (version 2.2.6)

begin model

begin parameters

# The following parameters were fit to experimental degranulation data using
# BioNetFit 1.0
# For more information, see the BioNetFit configuration file model_fit.conf

X_tot__FREE__6.13331088e+00
k_Xoff__FREE__1.91394890e-06
k_Xon__FREE__9.39816993e+04
kase__FREE__3.76143208e+00
kdegX__FREE__3.19130252e-04
kdegran__FREE__188893.283626392
km_Ship1__FREE__1.43154204e-03
km_Syk__FREE__2.87783197e-01
km_x__FREE__1.12185442e-01
koff__FREE__4.45671503e-03
kp_Ship1__FREE__1.10810534e+04
kp_Syk__FREE__2.65462642e+05
kp_x__FREE__7.81553987e+05
kpten__FREE__0.00995093271320638
ksynth1__FREE__1.84930114e-02
pase__FREE__1.60206452e-01

# End fit parameters

# system size scaling factor (>0)
```

```
f 1 # cells per reaction compartment

# Avogadro constant
NA 6.02214e23 # molecules per mol

# time unit conversion factor
T 60 # s per min

# channel volume in microfluidic device
Vchannel 500e-9 # L per channel (500 nL)

# number of cells per channel
Nchannel 1000 # cells per channel

# volume of extracellular fluid
Vecf=f*(Vchannel/Nchannel) # L per cell (500 pL/cell)

# cytosolic volume of an RBL-2H3 cell
# This estimate corresponds to the volume of a sphere with a diameter of
# 17.9 microns.
Vcyt f*3e-12 # L per cell (3 pL/cell)

# multivalent antigen abundance in medium at condition 0
Ag_tot_0 0 # copies per cell (cpc)

# multivalent antigen abundance in medium at condition 1
Ag_conc1 10e-9 # M (10 nM)
Ag_tot_1=Ag_conc1*(NA*Vecf) # cpc

# antigen receptor abundance
R_tot f*3e5 # cpc (1 nM)

# Syk abundance
Syk_tot f*3e5 # cpc (0.166 uM)

# Ship1 abundance
Ship1_tot f*3e5 # cpc (0.166 uM)
```

```
# rate constant for antigen capture
kon 1e7*T/(NA*Vecf) # /M/s converted to /cpc/min
# We assume a diffusion-limited value of 1e7 /M/s

# rate constant for antigen release
koff koff__FREE__*T # 4.46e-3 /s converted to 0.268 /min

# rate constant for pseudo first-order phosphorylation of antigen-engaged
# receptor
kase kase__FREE__*T # 3.76 /s converted to 2.25e+2 /min

# rate constant for pseuedo first-order dephosphorylation of phosphorylated
# receptor
pase pase__FREE__*T # 1.60e-1 /s converted to 9.6 /min

kp_Syk kp_Syk__FREE__*T/(NA*Vcyt) # 2.65e+5 /M/s converted to 8.80e-6 /cpc/min
km_Syk km_Syk__FREE__*T # 2.88e-1 /s converted to 17.2 /min

kp_Ship1 kp_Ship1__FREE__*T/(NA*Vcyt) # 1.11e+4 /M/s converted to
# 3.68e-7 /cpc/min
km_Ship1 km_Ship1__FREE__*T # 1.43e-3 /s converted to 8.58e-2 /min

ksynth1 ksynth1__FREE__*T # 1.85e-2 /s converted to 1.11 /min
kdeg1 1e7*T/(NA*Vcyt) # /M/s converted to /cpc/min;
# We assume a diffusion-limited value of 1e7 /M/s

# Rate of basal degradation of PIP3 and/or IP3
kpten kpten__FREE__*T # 9.95e-3 /s converted to 0.597 /min


H_tot 1e6 # cpc

kdegran kdegran__FREE__*T/(NA*Vcyt) # 1.89e+5 /M/s converted to 6.27e-6/cpc/min

# rate constant for degradation of X
# (in one step, including ubiquitination and proteosomal degradation)
kdegX kdegX__FREE__*T # 3.19e-4 /s converted to 1.91e-2 /min
```

```
k_Xon k_Xon__FREE__*T/(NA*Vcyt) # 9.40e+4 /M/s converted to 3.12e-6 /cpc/min
k_Xoff k_Xoff__FREE__*T # 1.91e-6 /s converted to 1.15e-4 /min

kp_x kp_x__FREE__*T/(NA*Vcyt) # 7.81e+5 /M/s converted to 2.59e-5 /cpc/min
km_x km_x__FREE__*T # 1.12e-1 /s converted to 7.26 /min

# abundance of hypothetical Ship1 cofactor X
X_tot=X_tot__FREE__*Ship1_tot # 6.13 times the Ship1 concentration, converted
# to 1.84e6 cpc

end parameters

begin molecule types

# antigen (DNP-conjugated BSA)
# DNP: 2,4-dinitrophenyl groups
Ag(DNP)

# antigen receptor (anti-DNP IgE bound to FcεRI)
# IgE: anti-DNP IgE
# Yb:  tyrosine residues (0, unmodified; P, phosphorylated)
# in the β chain ITAM of FcεRI
# Yg:  tyrosine residues (0, unmodified; P, phosphorylated)
# in the γ chain ITAMs of FcεRI
R(IgE,Yb~0~P,Yg~0~P)

# protein tyrosine kinase Syk
# tSH2:  tandem SH2 domains
Syk(tSH2)

# lipid phosphatase Ship1
# SH2:  Src homology 2 (SH2) domain
# x:  binding site for hypothethical Ship1 cofactor X
Ship1(SH2,x)

# hypothetical Ship1 cofactor X
# s:  Ship1 binding site (on, active; off, inactive)
```

```
X(s~on~off)

# phosphatidylinositol (3,4,5)-trisphosphate
PIP3()


# β-hexosaminidase
# loc:  location (in, inside granules; out, secreted/outside cell)
H(loc~in~out)

end molecule types

begin seed species

# initial conditions

# initial abundance of free antigen (in medium) per cell
# The $ prefix indicates that this abundance is to be held constant.
$Ag(DNP) Ag_tot_1

# initial abundance of free antigen receptor
R(IgE,Yb~0,Yg~0) R_tot

# initial abundance of (inactive) cytosolic Syk
Syk(tSH2) Syk_tot

# initial abundance of (inactive) cytosolic Ship1
Ship1(SH2,x) Ship1_tot

# initial abundance of PIP3
PIP3() 0

# initial abundance of β-hexosaminidase (stored in cellular granules)
H(loc~in) H_tot

# initial abundance of inactive hypothetical Ship1 cofactor X
X(s~off) X_tot
```

```
end seed species

begin observables

# simulation outputs

Molecules Ag_total Ag() # This quantity may accumulate because free Ag
# abundance is held fixed.
Molecules Ag_free Ag(DNP) # This quantity is held constant, except for
# adjustments in the actions block.
Molecules R_bound R(IgE!+) # number of antigen-bound receptors per cell
Molecules R_free R(IgE) # number of antigen-free receptors per cell
Molecules RP R(Yg~P!?)  # number of phosphorylated receptors per cell
Molecules R0 R(Yg~0) # number of unphosphorylated receptors per cell
Molecules actSyk Syk(tSH2!+) # number of Syk molecules recruited to antigen
# receptors
Molecules actShip1 Ship1(SH2!+,x!+) # number of Ship1 molecules recruited to
# antigen receptors and bound to X
Molecules Ship1_total Ship1() # total abundance of Ship1
Molecules PIP3_total PIP3() # total abundance of PIP3
Molecules degranulation H(loc~out) # abundance of secreted β-hexominadase
# (surrogate for secreted mediators of inflammation)
Molecules Xall X() # total abundance of hypothetical Ship1 cofactor X
Molecules X_on_free X(s~on) # abundance of free X in activated state
Molecules X_on_free_or_bound X(s~on!?)  # abundance of X (bound or free) in
# activated state
Molecules XShip1 X(s~on!1).Ship1(x!1) # abundance of Ship1 bound to (activated)
# cofactor X

end observables

begin reaction rules

# ligand-receptor binding
# As a simplification, we consider a one-step binding mechanism.
# Thus, the rate constants are effective parameters that reflect
# capture and release of (multivalent) antigen as well as the effects
# of antigen-mediated receptor aggregation on the residence time of antigen
```

```
# on the cell surface.  Recall that the antigen receptor is a long-lived 1:1
# complex of antigen-specific IgE and FcεRI, the high-affinity Fc
# receptor for IgE.
Ag(DNP)+R(IgE)<->Ag(DNP!1).R(IgE!1) kon,koff

# ligand-dependent receptor phosphorylation
# We assume that bound receptors are competent for (Lyn-mediated)
# phosphorylation of the tyrosines in β and γ ITAMs.
# As a simplification, we assume that the β and γ sites are
# phosphorylated simultaneously as part of a single process.
# The effective rate constant for phosphorylation is assumed to capture
# associaton of Lyn with receptors and the relationship between the abundance
# of cell-associated antigen and the extent of antigen-mediated receptor
# crosslinking.
R(IgE!+,Yb~0,Yg~0)->R(IgE!+,Yb~P,Yg~P) kase

# receptor dephosphorylation
# # As a simplification, we assume that the β and γ sites are
# dephosphorylated simultaneously as part of a single process.
# As an additional simplification, we consider a pseudo first-order mechanism.
# Thus, the effective rate constant for phosphorylation can be viewed
# as the Vmax/KM ratio for phosphatases.  We expect phosphatase activity to be
# high.
R(Yb~P,Yg~P)->R(Yb~0,Yg~0) pase

# recruitment of Syk to the γ subunit of the phosphorylated receptor
# As a simplification, we consider a one-step binding mechanism.
# We expect this simplification to be accurate even though the tandem
# SH2 domains of Syk dock to a doubly phosphorylated γ ITAM via a
# two-step mechanism because isomerization reactions are expected to be fast.
R(Yg~P)+Syk(tSH2)<->R(Yg~P!1).Syk(tSH2!1) kp_Syk,km_Syk

# recruitment of Ship1 to the β subunit of the phosphorylated receptor
# The SH2 domain Ship1 interacts with the phosphorylated β ITAM.
R(Yb~P)+Ship1(SH2)<->R(Yb~P!1).Ship1(SH2!1) kp_Ship1,km_Ship1

# receptor-mediated activation of hypothetical Ship1 cofactor X
# As a simplification, we consider a pseudo second-order mechanism.
```

```
# The rate constant for activation of X can be viewed as the kcat/KM ratio for
# a receptor-associated kinase responsible for activating phosphorylation of X.
# Note that our choice to check the phosphorylation state of R(Yb) rather than
# R(Yg) is arbitrary, as within the simplifications of this model, the two
# sites are phosphorylated and dephosphorylated simultaneously.
R(Yb~P!?)+X(s~off)->R(Yb~P!?)+X(s~on) k_Xon

# decativation of X
# As a simplification, we consider a pseudo first-order mechanism.
X(s~on)->X(s~off) k_Xoff

# activated Syk-dependent synthesis of PIP3
# PI3K is recruited to phosphorylated LAT, a key substrate of Syk and plasma
# membrane protein, and there generates PI(3,4,5)P3 from PI(4,5)P2.
# As a simplification, we assume that PI3K activity (and PIP3 generation) is
# proportional to the abundance of receptor-recruited Syk.  Thus, we assume that
# the rate constant for PIP3 generation captures the relationship between Syk
# recruitment/activation and PI3K recruitment/activation enabled by
# Syk-mediated phosphorylation of LAT.
Syk(tSH2!+)->Syk(tSH2!+)+PIP3() ksynth1

# interaction of activated X with Ship1
# We assume that X-Ship1 interaction requires prior receptor-mediated
# activation of X. Thus, only X with a Ship1 binding site "s" in the "on" state
# is allowed to associate with Ship1.
X(s~on)+Ship1(x)<->X(s~on!1).Ship1(x!1) kp_x,km_x


# activated Ship1-dependent clearance of PIP3
# We assume that membrane-associated Ship1, when bound to both the receptor and
# a cofactor (X), is mainly responsible for clearance of PIP3.  Ship1 catalyzes
# the conversion of PI(3,4,5)P3 to PI(3,4)P2.
Ship1(SH2!+,x!+)+PIP3()->Ship1(SH2!+,x!+) kdeg1

#clearance of PIP3
# PIP3 is degraded to PI(4,5)P2 by PTEN.
# We assume that all degradation of PIP3 can be simplified into a single
# pseudo first-order process.
```

```
PIP3()->0 kpten


# Degradation of activated X
# We assume that activated X is subject to ubiquitination, followed by
# degradation in the proteosome
# We take this to occur via a pseudo first-order process
# X bound to Ship1 is also subject to proteosomal degradation, which is assumed
# to liberate Ship1.
# We assume that synthesis and degradation of *inactive* X are sufficiently
# slow to not be relevant on the time scales studied.
X(s~on)->0 kdegX
X(s~on!1).Ship1(x!1)->Ship1(x) kdegX

# Degranulation due to the presence of PIP3
# PIP3 serves as a PLCγ substrate, leading to the production of IP3,
# stimulating store-operated calcium release followed by degranulation
# We assume this occurs as a pseudo second order process.
PIP3()+H(loc~in)->PIP3()+H(loc~out) kdegran

end reaction rules

end model

begin actions

# The command below instructs BioNetGen to derive a reaction network from the
# model's rules.
# The output is sent to a file that has a .net filename extension.
# The .net file can be further processed to generate the ordinary differential
# equations corresponding to the reaction network and the rate laws associated
# with the model's rules.
generate_network(overwrite=>1)

# The commands below illustrate how we simulated
# 1) an initial period of multivalent antigen-stimulated IgE receptor
# signaling,
# 2) an intermediate period of monovalent antigen-induced IgE receptor
```

```
# signaling quiescence, and
# 3) a second and final period of multivalent antigen-stimulated IgE receptor
# signaling.

# For the purposes of fitting to experimental data, we repeat the series of 3
# simulations for each time delay that was tested experimentally
# For each one, a .exp file was saved containing the appropriate experimental
# data point from Fig.  4
# BioNetFit was used to fit the model to the .exp files.

# first 5-min period of multivalent antigen stimulation
setConcentration("Ag(DNP)","Ag_tot_1")
simulate(suffix=>"p1_5",method=>"ode",t_end=>5,n_steps=>50)
# Remember these concentrations, so we can go back for each interval tested
saveConcentrations()

# treatment with monovalent antigen to rapidly induce signaling quiescence
setConcentration("Ag(DNP)","Ag_tot_0")
# Interval is 5 min
simulate(suffix=>"p2_5",method=>"ode",t_end=>5,n_steps=>50)

# second 5-min period of multivalent antigen stimulation
setConcentration("Ag(DNP)","Ag_tot_1")
# Remove all existing secreted β hex, in order to measure only what is
# secreted during this interval
setConcentration("H(loc~out)",0)
simulate(suffix=>"p3_5",method=>"ode",t_end=>5,n_steps=>50)

# Repeat for an interval of 30 min
resetConcentrations() # Revert to the state after simulation 1
setConcentration("Ag(DNP)","Ag_tot_0")
simulate(suffix=>"p2_30",method=>"ode",t_end=>30,n_steps=>300)
setConcentration("Ag(DNP)","Ag_tot_1")
setConcentration("H(loc~out)",0)
simulate(suffix=>"p3_30",method=>"ode",t_end=>5,n_steps=>50)


# Repeat for an interval of 60 min
```

```
resetConcentrations() # Revert to the state after simulation 1
setConcentration("Ag(DNP)","Ag_tot_0")
simulate(suffix=>"p2_60",method=>"ode",t_end=>60,n_steps=>600)
setConcentration("Ag(DNP)","Ag_tot_1")
setConcentration("H(loc~out)",0)
simulate(suffix=>"p3_60",method=>"ode",t_end=>5,n_steps=>50)

# Repeat for an interval of 120 min
resetConcentrations() # Revert to the state after simulation 1
setConcentration("Ag(DNP)","Ag_tot_0")
simulate(suffix=>"p2_120",method=>"ode",t_end=>120,n_steps=>1200)
setConcentration("Ag(DNP)","Ag_tot_1")
setConcentration("H(loc~out)",0)
simulate(suffix=>"p3_120",method=>"ode",t_end=>5,n_steps=>50)

# Repeat for an interval of 240 min
resetConcentrations() # Revert to the state after simulation 1
setConcentration("Ag(DNP)","Ag_tot_0")
simulate(suffix=>"p2_240",method=>"ode",t_end=>240,n_steps=>2400)
setConcentration("Ag(DNP)","Ag_tot_1")
setConcentration("H(loc~out)",0)
simulate(suffix=>"p3_240",method=>"ode",t_end=>5,n_steps=>50)

end actions
```
